# Epigenetic regulation in metabolic diseases: mechanisms and advances in clinical study

**DOI:** 10.1038/s41392-023-01333-7

**Published:** 2023-03-02

**Authors:** Yan-Lin Wu, Zheng-Jun Lin, Chang-Chun Li, Xiao Lin, Su-Kang Shan, Bei Guo, Ming-Hui Zheng, Fuxingzi Li, Ling-Qing Yuan, Zhi-hong Li

**Affiliations:** 1 National Clinical Research Center for Metabolic Disease, Department of Metabolism and Endocrinology, The Second Xiangya Hospital, Central South University, Changsha, Hunan 410011 China; 2 Department of Orthopaedics, The Second Xiangya Hospital, Central South University, Changsha, Hunan 410011 China; 3 Hunan Key Laboratory of Tumor Models and Individualized Medicine, The Second Xiangya Hospital, Central South University, Changsha, Hunan 410011 China; 4 Department of Radiology, The Second Xiangya Hospital, Central South University, Changsha, Hunan 410011 China

**Keywords:** Epigenetics, Endocrine system and metabolic diseases

## Abstract

Epigenetics regulates gene expression and has been confirmed to play a critical role in a variety of metabolic diseases, such as diabetes, obesity, non-alcoholic fatty liver disease (NAFLD), osteoporosis, gout, hyperthyroidism, hypothyroidism and others. The term ‘epigenetics’ was firstly proposed in 1942 and with the development of technologies, the exploration of epigenetics has made great progresses. There are four main epigenetic mechanisms, including DNA methylation, histone modification, chromatin remodelling, and noncoding RNA (ncRNA), which exert different effects on metabolic diseases. Genetic and non-genetic factors, including ageing, diet, and exercise, interact with epigenetics and jointly affect the formation of a phenotype. Understanding epigenetics could be applied to diagnosing and treating metabolic diseases in the clinic, including epigenetic biomarkers, epigenetic drugs, and epigenetic editing. In this review, we introduce the brief history of epigenetics as well as the milestone events since the proposal of the term ‘epigenetics’. Moreover, we summarise the research methods of epigenetics and introduce four main general mechanisms of epigenetic modulation. Furthermore, we summarise epigenetic mechanisms in metabolic diseases and introduce the interaction between epigenetics and genetic or non-genetic factors. Finally, we introduce the clinical trials and applications of epigenetics in metabolic diseases.

## Introduction

Metabolic diseases are a growing worldwide health challenge due to their dramatically increasing incidence.^1,2^ These diseases include obesity,^3^ type 2 diabetes (T2D),^4^ nonalcoholic fatty liver disease (NAFLD),^5^ osteoporosis,^6^ gout,^7^ hyperthyroidism^8^ and hypothyroidism.^9^ Diabetes has become the ninth major cause of death worldwide. According to the statistics of the International Diabetes Federation (IDF),^10^ 537 million adults had diabetes in 2021, of which more than 90% had T2D. The number is estimated to increase to 783 million by 2045. Besides, obesity has become a primary public health problem globally and a dramatically increasing prevalence of overweight and obesity has also been observed during the past decades. More than 1.9 billion adults and over 650 million adults were obese or overweight, respectively, around the world in 2016, which accounted for approximately 39% of the global population.^11^ The most recent national survey based on the Chinese population showed that 34.3% of adults were overweight and 16.4% of adults were obese.^12^ With a global prevalence of 25%, NAFLD has become the most common chronic liver disease worldwide.^13^ It is estimated that in 2019, the global prevalence of NAFLD in Asia was 29.62%.^14^ In addition, gout is the most common category of inflammatory arthritis caused by the deposition of monosodium urate (MSU) crystals in articular and non-articular structures, with a prevalence of 1–4% and an incidence of 0.1%–0.3% worldwide.^15^ These data indicate that metabolic diseases are a severe burden in human society owing to the ensuing high morbidity and mortality; hence, uncovering the mechanisms and therapeutics of metabolic diseases is essential.

The underlying mechanisms of metabolic diseases are multifaceted, and both genetic and non-genetic factors are critically responsible for the initiation and development of metabolic diseases.^1,16^ Emerging evidence indicates that epigenetic regulation plays a crucial role in the occurrence and progression of diverse metabolic diseases.^16–21^ Epigenetics is regarded as various covalent modifications of nucleic acids and histone proteins which regulate gene function and expression and the chromatin structure cooperatively.^22–24^ Epigenetic regulation can occur at various levels, including through DNA methylation, histone modifications, chromatin remodelling, and noncoding RNA (ncRNA) modulation.^24–26^ Epigenetics is fundamental to several biological processes, such as cell differentiation, replication, and adhesion.^27–29^ Notably, multiple epigenetic modifications are significantly correlated with metabolic disease-related gene function and expression and often occur early in diseases, thus exhibiting promising potential as clinical biomarkers for patients with metabolic diseases.^30–32^ Epigenetic-based diagnostic and therapeutic efficacy prediction and evaluation tools greatly contribute to precision medicine in metabolic diseases.^21,33,34^ Moreover, epigenetic regulation is reversible and dynamically modulated, meaning that epigenetic-related changes to genes and proteins could serve as novel therapeutic targets in clinical settings.^17^ Therefore, deciphering the epigenetic regulation of metabolic diseases is crucial to understand metabolic diseases initiation and progression, and to develop novel preventive or curative therapeutic strategies in clinical metabolic disease management.

In this review, we introduce the history and four general mechanisms of epigenetic modulation and systematically summarise recent progress regarding the roles of epigenetic regulation in metabolic diseases as well as the underlying mechanisms. Besides, we discuss the clinical applications of epigenetic regulation as promising epigenetic biomarkers and novel therapeutic targets in metabolic disease treatment.

## Overview of epigenetics

### A brief history of epigenetics

In 1942, the English developmental biologist Conrad Hal Waddington proposed the new term ‘epigenetics’ as the processes by which the genotype brings the phenotype into being.^35^ Moreover, in 1957, Waddington published his famous drawing of the ‘epigenetic landscape’, which suggested that the process of cellular differentiation may be regulated by changes in the ‘epigenetic landscape’ instead of alterations in genetic inheritance.^36^ DNA modifications were discovered in 1948,^37^ and in 1975, Holliday et al.^38^ illustrated that DNA methylation are involved in gene regulation, particularly 5-methylcytosine (5mC). Furthermore, in 1980, Razin et al.^39^ found that DNA methylation represses gene function and differentiation. In 1964, histone modifications, especially acetylation, were described for the first time, and researchers discovered their close relationship with the regulation of RNA synthesis.^40^ The currently model of the nucleosomal organisation of chromatin was proposed in 1974.^41^ The model describes that the basic unit of chromatin is the nucleosome particle, which consists of four histones (histone octamers) and 147 base pairs of DNA wrapped around them. In 1976, Sanger^42^ first discovered circular RNA (circRNA) molecules in Viroids. H19 was identified as the first long ncRNA (lncRNA) involved in epigenetic regulation in 1990.^43^ In 1994, the first microRNA (miRNA), lin-4, was discovered in the nematode Caenorhabditis elegans by Lee and colleagues.^44^ In 1997, the crystallographic structure of the nucleosome core particle of chromatin was visualised by X-ray.^45^ In 1996, the first nuclear histone acetyltransferase (HAT) and the first histone deacetylase (HDAC) were discovered separately.^46,47^

Since the beginning of the 21st century, epigenetics has developed rapidly, and there has been a tremendous amount of research published. In 2000, SUV39H1 was discovered as the first histone lysine methyltransferase (KMT), which selectively trimethylates histone H3 lysine 9 (H3K9me3).^48^ In 2004, the first histone lysine demethylase (KDM), LSD1, was discovered. In 2006, the first wave of epigenetic drugs, including decitabine and vorinostat, was approved by the U.S. Food and Drug Administration (FDA) and was used to treat human cancers. In 2012, oncohistones were first reported as mutations in histone genes, which were related to cancer.^49,50^ In 2015, the U.S. National Institutes of Health (NIH) Roadmap Epigenomics Consortium published 111 human reference epigenomes.^51^

**Fig. 1:**
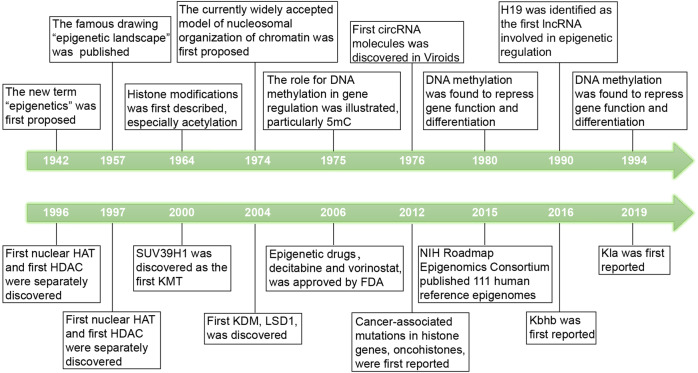
The milestone events related to epigenetics. Key discoveries are highlighted

In less than 100 years, the concept of epigenetics has developed rapidly. We summarise the milestone events related to epigenetics in Fig. 1.

### Methods to study epigenomic and epigenetic states

There are growing interests in the functions of epigenomics and the related molecular mechanisms. Thus, the development of new technologies contributes to providing a better understanding of epigenomics (Table 1).

**Table 1:** Methods to study epigenomic and epigenetic states

Methods	Purposes
ChIP-seq	Studying protein/DNA-binding and histone-modification sites in a genome-wide manner
ISH-PLA	Detecting histone modifications at specific gene loci in single cells
DNase-seq	Mapping Deoxyribonuclease I hypersensitive sites (DHSs)
ATAC-seq	Sequencing regions of loosely packaged chromatin through transposition of markers
FAIRE-Seq	Sequencing the DNA unbound by chromatin proteins
MNase-seq	sequencing regions of DNA bound by histones or other chromatin-bound proteins
BS-Seq	5mC detection
oxBS-Seq	Quantitative mapping of 5hmC
fCAB-Seq	Sequencing 5fC
CAB-Seq	Mapping 5caC
CUT&TAG	Analyzing protein interactions with DNA
CUT&RUN	Analyzing protein interactions with DNA

Chromatin immunoprecipitation followed by sequencing (ChIP-seq) analysis is a useful tool to study protein/DNA-binding and histone-modification sites in a genome-wide manner, which provides genome-wide and locus-specific modification profiles and temporal factor occupancy. The general principle is to fix the interaction in the DNA-protein complexes by using a crosslinking agent such as formaldehyde, then cut the cross-linked chromatin into fragments as small as 200–600 base pairs, and use a specific antibody targeted to the protein to precipitate the DNA-protein complex. After reversing the cross-linking, the immunoprecipitated DNA fragments are purified, sequenced, and mapped to the genome to locate the site of interaction relative to a gene’s transcription start site (TSS).^52,53^ However, there are still some drawbacks to ChIP-seq analysis. It does not provide single-cell resolution in heterogeneous cell populations and lacks of spatial resolution. In situ hybridisation and proximity ligation assays (ISH-PLA) are used to detect histone modifications at specific gene loci in single cells through proximity ligation assays and in situ hybridisation.^54^ However, ISH-PLA is highly antibody-dependent and has not been widely used.

There are some methods to evaluate chromatin accessibility. Deoxyribonuclease I (DNase I)-hypersensitive site sequencing (DNase-seq) is a method to determine chromatin accessibility and its underlying regulatory lexicon.^55^ However, the need for a great number of cells, typically in the tens of millions, limits this approach. Compared with DNase-seq, the assay for transposase-accessible chromatin using sequencing (ATAC-seq) is a simple method to map genome-wide chromatin accessibility or open chromatin landscape, an approach that requires a relatively small number of cells. However, ATAC-seq is difficult to detect nucleosome as low read coverage beyond peaks is typical. Moreover, the analysis of ATAC-seq results is limited by the bioinformatics analysis.^56^ Besides, formaldehyde-assisted isolation of regulatory elements (FAIRE) analysis coupled with deep sequencing (FAIRE-Seq) is also a useful tool to identify open chromatin regions.^57^ But the result FAIRE-Seq is difficult to interpret with the high background and low signal-to-noise ratio. Micrococcal nuclease sequencing (MNase-seq) is an indirect method to evaluate chromatin accessibility and has been used for mapping nucleosome positions at individual genes.^58^ However, MNase sites might not account for the entire genome and AT-dependent sequence bias may exist in MNase-seq.

Several high-throughput detection strategies have been developed to study DNA and RNA modifications. Different modifications have different sequencing assays. Bisulfite sequencing (BS-Seq) is commonly utilised for 5mC detection.^59^ However, BS-Seq is difficult to discriminate between 5mC and 5-hydroxymethylcytosine (5hmC). Oxidative bisulfite sequencing (oxBS-Seq) is developed for quantitative mapping of 5hmC.^60^ 5-Formylcytosine (5fC) chemically assisted bisulfite sequencing (fCAB-Seq) was the first quantitative method to sequence 5fC,^61^ while mapping 5-carboxylcytosine (5caC) uses chemical modification-assisted bisulfite sequencing (CAB-Seq).^62^

Cleavage Under Targets and Tagmentation (CUT&TAG) and Cleavage Under Targets and Release Using Nuclease (CUT&RUN) are novel techniques based on antibodies. CUT&TAG offers high-resolution sequencing libraries for small samples and single cells.^63^ Single-cell CUT&TAG has been used to analysis transcription factors and histone modifications in complex tissues.^64^ CUT&RUN is a new strategy to map protein-DNA interactions in situ, which is cost-effective and easy to perform.^65^

In summary, the technologies associated with epigenetics have progressed rapidly. As each approach has its pros and cons, multiple methods are applied simultaneously to study epigenomic and epigenetic states. Due to the need for epigenetic research, it is necessary to develop simpler and more practical technologies.

## Epigenetic regulatory mechanisms

**Fig. 2:**
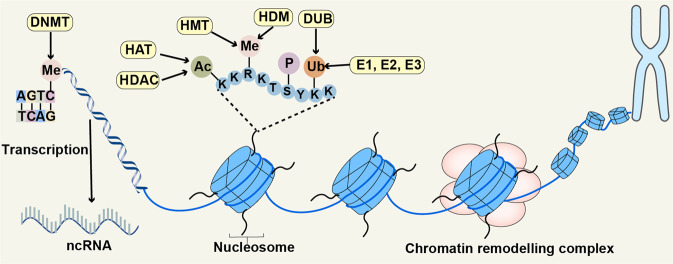
Four different epigenetic regulatory mechanisms. The figure presented DNA methylation, histone modification, chromatin remodelling, and ncRNAs. DNA methylation is a universal chemical modification by which methyl groups (Me) are added to the DNA molecule, usually happening on the CpG islands. Histone undergoes several different post-translational modifications, including acetyl (Ac), Me, phosphate (P) and ubiquitin (Ub). Chromatin remodelling complexes change the packaging state of chromatin by moving, sliding, disrupting, or restructuring the nucleosome. ncRNAs are participated in multiple physiological and pathological process by targeting different molecules. This figure was generated with Servier Medical Art (https://smart.servier.com/)

We will discuss four epigenetic regulatory mechanisms: DNA methylation, histone modification, chromatin remodelling, and ncRNA. All of them can alter gene expression without changing its sequence (Fig. 2).

### DNA methylation

DNA methylation is a universal chemical modification by which methyl groups are added to the DNA molecule. DNA methylation most often happens on the cytosine phosphate guanine (CpG) islands, a site in which a cytosine is located next to a guanidine.^66^ Mainly noted within telomeres, centromeres, repeat sequences, and inactive X-chromosomes, DNA methylation is involved in several biological processes, such as genomic imprinting, regulation of epigenetic gene expression, genome stability and transposon silencing.^67,68^ Studies have revealed multiple forms of DNA methylation, including 5mC, 5hmC, 5fC, and 5caC.^69–71^ 5mC is the common epigenetic modification in the human genome and has been well studied. In contrast, the other forms of DNA methylation are relatively rare.

DNA methyltransferases (DNMTs) are responsible for DNA methylation, which transfer a methyl group from the *S*-adenosylmethionine (SAM) to the 5′-site of the cytosine ring in DNA. In the human genome, five DNMTs have been identified, including DNMT1, DNMT2, DNMT3A, DNMT3B, and DNMT3L. Although DNMT2 and DNMT3L have sequence conservation with the other three DNMTs, they do not possess catalytic activity.^72^ DNMTs can be divided into two groups, namely *de novo* DNMTs and maintenance DNMTs. Maintenance DNMTs only include DNMT1, which is involved in maintaining already established DNA methylation marks. DNMT3A and DNMT3B belong to *de novo* DNMTs and they are involved in establishing a new DNA methylation pattern at previously unmethylated sites.^73^

### Histone modification

As a component of octamer, histone undergoes several different post-translational modifications through different histone-modifying enzymes.^25^ There are various types of histone modifications, such as acetylation, methylation, lactylation, phosphorylation, dopaminylation, and ubiquitination, among others.^40,74–76^ Histone modifications not only remove or add binding sites in specific protein complexes, but also affect the interactions of histone and DNA or various histones, thereby regulating gene expression. Until now, most studies on histone modification have focused on histone acetylation.

#### Histone acetylation

Histone acetylation mostly occurs at the N-terminus of H3 and H4 of lysine. There are more than 40 different lysine sites modified by acetylation.^77^ Histone acetylation is a reversible post-translational modification that has been well researched. This modification is mainly modulated by HATs and HDACs.^78^

HATs promote histone acetylation by catalysing the transfer of an acetyl group to a lysine site. HATs are mainly divided into three families: including P300 and cyclic adenosine monophosphate (AMP) response element-binding protein (CBP) complex, MYST (namely MOZ, Ybf2/Sas3, Sas2, and Tip60), and GCN5-related *N*-acetyltransferase (GNAT).^79^ The GNAT family includes HAT1, GCN5, and PCAF. Notably, the CBP-P300 complex functions in concert with other HATs, such as PCAF.^80^

On the other hand, HDACs inhibit histone acetylation by catalysing acetyl group removal. HDACs have been classified into four classes. There are four HDACs in Class I, including HDAC1, HDAC2, HDAC3, and HDAC8, which are RPD3-like proteins and widely distributed in the nucleus of human cell lines and tissues. There are two subclasses in Class II HDACs with tissue-specific expression, HDAC4, HDAC5, HDAC7, and HDAC9 belong to Class IIa, while Class IIb includes HDAC6 and HDAC10. Class III is nicotinamide adenine dinucleotide (NAD + )-dependent and includes sirtuins (SIRT1–7). Finally, Class IV only includes HDAC11.^81,82^

Histone acetylation readers, mainly including bromodomains (BrDs), can read the acetylation marks on lysine residues. The first histone modification readers BrDs were reported in 1999.^83^ BrDs were evolutionarily conserved of approximately 110 amino acids. 61 BrDs were identified in 46 different human proteins in 2012, and they were classified into eight families according to the structure and sequence similarity.^84^ Present in different nuclear proteins, such as chromatin remodelling complexes, BrDs were responsible for chromatin remodelling and transcriptional regulation, thus, acting as possible targets for epigenetic drugs.^85,86^

#### Other histone modifications

Regulated by histone methyltransferases (HMTs) and histone demethylases (HDMs), histone methylation mainly occurs at the N-terminus of H3 and H4 of lysine or arginine residues.^87^ There is mono-, di-, or trimethylation at lysine residues, while arginine residues could be monomethylated or asymmetrically or symmetrically dimethylated. Methylation at different sites presents various effects – for example, transcriptional activation-related methylations exhibited on histone H3 on lysine 4 (H3K4), H3K36, H3K79, and arginine 17 (H3R17).^88–91^ On the contrary, transcriptional repression of histone methylation is observed on H3K9/27 or H4K20.^92–94^ Histone methylation of lysine is regulated by KMTs and erased by KDMs, while protein arginine methyltransferases (PRMTs) catalyse histone arginine methylation.^95^

Histone ubiquitination is quite different from other histone modifications due to the covalent binding of a 76–amino acid protein, which is regulated by ubiquitination enzymes and deubiquitinating enzymes (DUBs).^96^ Histone ubiquitination occurs at H1, H2A, H2B, H3, and H4, which is involved in the process of genotoxic stress, DNA damage response (DDR), and transcriptional regulation.^97–99^ Histone phosphorylation usually occurs at H3 or H2A of serine, threonine, and tyrosine, which is related to centromere function, chromosome condensation, and transcriptional activation.^100–102^

Histone lysine β-hydroxybutyrylation (Kbhb) was first reported in 2016, whose levels were significantly elevated under conditions of diabetic ketosis or starvation.^103^ Kbhb is catalysed by p300, while SIRT1 to SIRT3 and HDAC1 to HDAC3 remove Kbhb.^104^ In addition, p300 could catalyze lysine propionylation (Kpr), butyrylation (Kbu), crotonylation (Kcr) in histones.^105,106^ Histone lysine lactylation (Kla) is a novel histone mark which was first reported in 2019. Kla is induced by lactate and p300 acts as a potential Kla writer protein.^107^

### Chromatin remodelling

Nucleosomes consist of histone protein octamers wrapped by DNA.^108^ As a general gene repressor, a nucleosome inhibits the initiation of transcription. Chromatin remodelling complexes can regulate gene expression by utilising the energy of adenosine triphosphate (ATP) hydrolysis to change the packaging state of chromatin by moving, sliding, disrupting, or restructuring the nucleosome.^109^ The remodelling process includes the dissociation of genomic DNA at the edge of the nucleosome with the formation of DNA protuberances on the surface of the histone octamer, the wavy propagation of the DNA ring on the surface of the nucleosome, and the repositioning of DNA without changing the total number of histone-DNA contacts.

There are four families of chromatin remodelling complexes, including the switching defective/sucrose nonfermenting (SWI/ SNF) family of remodellers,^110^ the imitation switch (ISWI) family of remodellers,^111^ the chromodomain helicase DNA binding (CHD) family of remodellers,^112^ and the inositol requiring 80 (INO80) family of remodellers.^113^ The SWI/SNF complex is composed of ATPase, actin-related protein (ARP), and body modules, and the three parts are separately associated with coupling ATP hydrolysis to DNA translocation, helping and linking the ATPase and the body module, and adding additional interactions with DNA- and histone- interacting subunits.^114^ The ISWI family of remodellers, also consist of three parts, including a regulatory auto-inhibition domain, a C-terminal hand-sant-slide (HSS) domain and an N-terminal RecA-like helicase domain. The RecA-like helicase domains form the ATPase domain and the HSS domain is responsible for nucleosome substrate binding. The CHD family of remodellers bind to chromatin-modifying and elongation factors, and histone acetylation inhibits the activity of the ISWI and CHD remodelling complexes. Similar to SWI/SNF, INO80 is composed of ATPase, ARP, and body modules. However, INO80 has a more extensive DNA-binding interface.^115,116^

### ncRNA

Numerous ncRNAs have been discovered as a result of the marked progress in sequencing technology. Only approximately 2% of the human genome can be translated into proteins, and the rest is transcribed into ncRNAs with diverse sizes and functions.^117^ According to their length, ncRNAs are mainly classified into small ncRNAs (sncRNAs, 18∼200 nucleotides), lncRNAs (>200 nucleotides),^118^ and circRNAs.^119^ Furthermore, sncRNAs can also be divided into miRNAs,^120^ small nuclear RNAs (snRNAs) and piwi-interacting RNAs (piRNAs). ncRNAs are responsible for multiple biological processes, such as apoptosis, autophagy^121^ and cellular proliferation.^122^ Moreover, ncRNAs are good diagnostic and prognostic biomarkers in various diseases, including metabolic diseases.^123^

#### miRNA

miRNAs are a vital type of endogenous RNAs with approximately 23 nucleotides in length, which originate from a double-stranded or hairpin RNA precursor. RNA polymerase II contributes to the biogenesis of miRNAs.^124^ miRNAs can inhibit gene expression and suppress translation by incorporating RNA-induced silencing complex (RISC) and paring to the 3′-untranslated regions (3′-UTRs) of target mRNAs.^125^ There is an interaction between miRNA expression and epigenetic machinery, including a feedback loop between them.^126^ Different miRNAs are regulated by epigenetic mechanisms, including DNA methylation and histone modifications. Besides, miRNAs are involved in epigenetic processes by modulating key enzymes of epigenetic modifications, such as HDACs and DNMTs.^127,128^ miRNAs participate in many metabolic diseases, such as obesity and diabetes.^129,130^

#### lncRNA

lncRNAs are a family of ncRNAs longer than 200 nucleotides. Due to their different locations relative to protein-coding genes, lncRNAs are grouped into five different classes: long intergenic non-coding RNAs (lincRNAs), antisense RNA, sense overlapping RNA, sense intronic RNA, and processed transcript ncRNA.^131^ lncRNAs participate in several crucial biological processes, such as regulating enzymatic activity and shaping chromosome structure, by acting as scaffolds, decoys or signals.^132^ Recently, lncRNAs is reported to act as miRNA sponges (in the cytoplasm) or host genes for the transcription of miRNAs (in the nucleus).^133^ In addition, lncRNAs mediate DNA methylation and act as modular scaffolds of histone modification complexes.^134,135^ Several lncRNAs are responsible for metabolic diseases, such as osteoporosis and diabetes mellitus.^136,137^

#### circRNA

circRNAs, a new class of endogenous RNAs containing covalently closed loop structures, are tissue and cell specific in eukaryotes.^138^ During the process of RNA splicing, circRNAs are generated from introns (intronic circRNAs or ciRNAs), exons (exonic circRNAs or ecircRNAs), or a combination of exons and introns (EIciRNAs).^139^ Many circRNAs play important biological roles in numerous metabolic diseases, including diabetes mellitus, by functioning as protein or miRNA sponges, and translating themselves.^140^ Current studies indicate that circRNAs participate in the regulation of DNA methylation and histone modification.^141,142^ circRNAs are involved in the process of metabolic diseases and have the potential to be as future therapeutics and disease biomarkers.^143^

## Epigenetic regulatory mechanisms in metabolic diseases

**Fig. 3:**
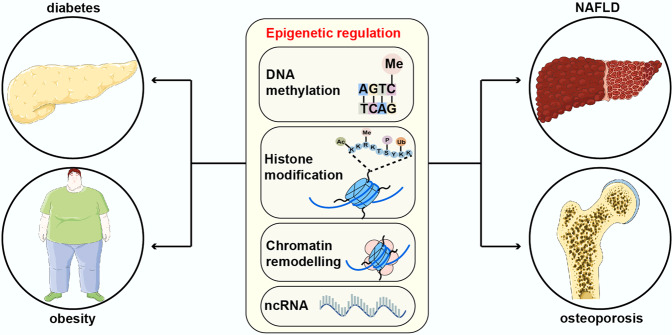
The roles of epigenetic regulation in metabolic diseases. The figure presented four main metabolic diseases where epigenetic regulation is involved, including diabetes and its complications, obesity, NAFLD and osteoporosis. This figure was generated with Servier Medical Art (https://smart.servier.com/)

Epigenetic regulation plays an indispensable role in numerous metabolic diseases, including diabetes mellitus and its complications, obesity, NAFLD, and osteoporosis (Fig. 3). A better understanding of epigenetic regulatory mechanisms in metabolic diseases helps us to know these diseases well, thereby providing novel therapies.

### The role of DNA methylation in metabolic disease

#### Diabetes mellitus and its complications

**Fig. 4:**
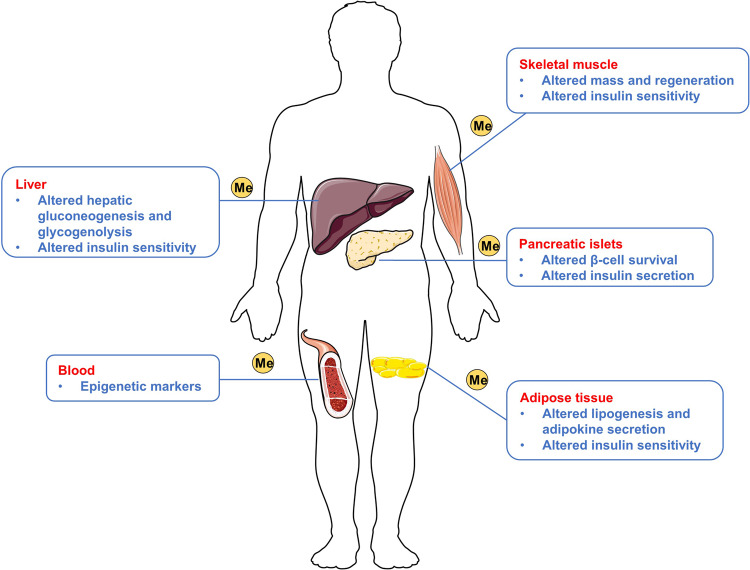
The different influence of DNA methylation in five human tissues for patients with T2D. The figure presented different influence of DNA methylation in patients with T2D in pancreatic islets, adipose tissue, skeletal muscle, liver and blood. This figure was generated with Servier Medical Art (https://smart.servier.com/)

There are changes in DNA methylation levels in organs and tissues related to the pathogenesis of T2D, such as pancreatic islets, adipose tissue, skeletal muscle, and liver (Fig. 4). In 2008, the first epigenetic study on T2D was conducted in pancreatic islets from patients with T2D. It is revealed that DNA methylation levels in the peroxisome proliferator-activated receptor gamma coactivator-1 α (PGC-1α) gene promoter was increased twofold in pancreatic islets of patients with T2D.^144^ Only one year later, researchers reported results on DNA methylation of the PGC-1α gene in skeletal muscle. They suggested that the PGC-1α promoter shows increased DNA methylation levels in patients with T2D, a finding consistent with the previous research. Furthermore, they found that the DNA methylation levels are negatively related to PGC-1α mRNA and mitochondrial DNA (mtDNA) in skeletal muscle.^145^ It has been acknowledged that the DNA methylation levels in the insulin promoter are elevated in patients with T2D;^146^ nevertheless, there are some hypomethylated CpG islands in these patients.^147^ In 2014, Dayeh and colleagues^148^ performed a genome-wide DNA methylation analysis of human pancreatic islets in patients with T2D. They revealed that regions further away from the TSS present greater methylation, while areas near the TSS in human islets are less methylated. Besides, the authors identified 1,649 CpG sites and 853 genes in T2D islets with changes in the DNA methylation level, including fat mass and obesity-associated (FTO), potassium voltage-gated channel subfamily Q member 1 (KCNQ1), and transcription factor-7-like-2 (TCF7L2).^148^ One study investigated DNA methylation levels in subcutaneous abdominal adipose tissue and identified 18 high-confidence candidate genes that are associated with diabetes, including cytoplasmic polyadenylation element-binding protein 4 (CPEB4) and fatty acid synthase (FASN).^149^ Krause et al.^150^ found decreased insulin receptor substrate 2 (IRS2) expression in the liver of patients with obesity and diabetes compared with participants with obesity but not diabetes. Decreased IRS2 expression is accompanied by DNA methylation at CpG5 in IRS2 and increased miRNA hsa-let-7e-5p (let-7e-5p) in liver.^150^

As blood is easily accessible, there are large number of studies highlighted DNA methylation levels changes in the blood cells. In 2020, García-Calzón et al.^151^ used genome-wide DNA methylation analysis in drug-naïve patients with diabetes and found that epigenetic markers in the blood cells can influence metformin tolerance and response. There were changes in DNA methylation in 11 sites in glycaemic responders compared with non-responders, while four sites showed different DNA methylation levels in metformin-tolerant patients versus intolerant patients. Furthermore, the risk of not responding to or not tolerating metformin increased with the DNA methylation levels.^151^ Recently, a large meta-analysis of individual epigenome-wide association studies (EWAS) was performed and explored DNA methylation in blood cells, including leucocyte, lymphocytes, monocytes and granulocytes, in patients with T2D. The authors identified three novel CpGs related to T2D in Europeans, including cg00144180, cg24704287, and cg16765088. They also discovered 77 T2D-associated differentially methylated regions (DMRs), most of which were hypomethylated in patients with T2D compared with the control groups.^152^

DNA methylation is also involved in diabetic complications. It is suggested that by decreasing the methylation levels of transforming growth factor-beta 1 (TGF-β1), ten-eleven translocation enzyme-2 (TET2) upregulated the expression of TGFβ1, which promoted the pathogenesis of diabetic kidney disease (DKD).^153^ The hypermethylation of cg04026387 and cg12869254 was participated in the progress of diabetic retinopathy (DR) and may act as new biomarkers for diagnosis DR.^154^ In addition, the expression of DNMT1 was upregulated induced by transient hyperglycemia, which hypermethylated angiotensin-1 (Ang-1) and decreased the expression of Ang-1, thus activating NF-κB and inhibiting the diabetic wound healing.^155^

Taken together, the upregulation or downregulation of DNA methylation levels occurs in several tissues and organs, exhibiting various impacts and resulting in the diabetes. In addition, it is convenient to collect blood and to detect alterations in DNA methylation levels.

#### Obesity

Body mass index (BMI) is widely used to measure the degree of obesity, which is calculated with height and weight. The relationship between DNA methylation and BMI has attracted attention from scientists, and several studies have been conducted. Sayols-Baixeras and his colleague performed an epigenome-wide association study and they validated 49 CpGs sites related to waist circumference and 94 CpGs related to BMI. Furthermore, they found new 33 CpGs sites associated with waist circumference and 70 CpGs related to BMI.^156^ In 2017, a large-scale study utilised 450k DNA methylation data from more than 10,000 whole blood samples and identified 187 CpG sites related to BMI. Besides, the result of genetic association analysis indicated that obesity is the cause of the alterations in DNA methylation levels, rather than the consequence.^157^ The lipid metabolism–related genes ATP-binding cassette subfamily G (WHITE) member 1 (ABCG1), carnitine palmitoyl-transferase 1 A (CPT1A), and sterol regulatory element-binding transcription factor 1 (SREBF1) show altered DNA methylation in obesity.^158–161^

Hypoxia develops in adipose tissue of patients with obesity.^162,163^ Dick et al.^164^ explored the relationship between DNA methylation levels and BMI by analysing whole-blood DNA. They identified five CpG sites, and three of the five CpG sites in the hypoxia-inducible factor 3 subunit alpha (HIF-3α) gene presented increased methylation, which is linked to increased BMI.^164^ In another study, the researchers analysed the relationship between obesity and DNA methylation in Chinese children. They found higher methylation levels in children with obesity at two sites, 46801699 and 46801642, in the HIF-3α gene. Moreover, the methylation levels were positively related to the alanine aminotransferase (ALT) levels, which is associated with the development of NAFLD.^165^

DNA methylation levels also reflect changes in weight. Bollepalli et al.^166^ found that the DNA methylation levels in subcutaneous adipose tissue (SAT) of participants with obesity were influenced by short- and long-term weight loss. They discovered that the expression of seven genes decreased during both short- and long-term weight loss, including BAG3, BHMT2, EPDR1, LEP, OSTM1, and UCHL1.^166^ Moreover, a clinical trial indicated that a specific DNA methylation signature in blood could reflect individual responsiveness to lifestyle intervention and methylation changes in the specific genes could predict successful weight loss.^167^ Besides, a randomised controlled trial suggested that DNA methylation in human adipose tissue could act as a predictor for weight increase during overfeeding in humans.^168^

Overall, DNA methylation is associated with the initiation and progression of obesity. It is not only associated with BMI, but also linked to hypoxia, and acts as a marker to reflect alterations in weight.

#### NAFLD

Diet plays a pivotal role in DNA methylation through several ways, including regulating the activity of enzymes associated with the one-carbon cycle and providing SAM as methyl donors.^169^ Recently, Chen et al.^170^ revealed that maternal consumption of a high-fat or high-cholesterol western diet can induce the pathogenesis of NAFLD in male offspring by modulating the expression of the apolipoprotein B (ApoB) gene. Based on DNA methylation analysis, they found that the ApoB gene promoter region presents increased methylation of CpG dinucleotides.^170^ Increased dipeptidyl peptidase 4 (DPP4) expression in the liver aggravates the development of NAFLD by autocrine and paracrine effects on hepatic insulin signalling and decreasing the levels of GLP-1.^171^ DNA methylation is also involved in DPP4-induced NAFLD. After feeding mice a high-fat diet (HFD) for 6 weeks, there was elevated DPP4 expression and reduced methylation levels of four CpG sites. In addition, by analysing human liver biopsy specimens from patients with obesity, the researchers found that DPP4 expression is positively correlated with the stages of hepatic steatosis and non-alcoholic steatohepatitis (NASH), while DNA methylation is negatively related to them. Moreover, DPP4 demethylation increases DPP4 expression early in life.^172^ Resveratrol (*trans*-3,5,4′-trihydroxystilbene), an inhibitor of glucose transporter 9 (GLUT9), regulates the methylation levels of NF-E2-related factor 2 (Nrf2) gene promoter to affect the development of NAFLD. Resveratrol can reverse Nrf2 promoter hypermethylation induced by high glucose (HG) and alleviates methylation levels of the Nrf2 promoter in the liver of mice induced by HFD, which is related to decreased triglyceride (TG) levels and downregulated expression of lipogenic genes, including Fas cell surface death receptor (FAS) and sterol regulatory element-binding protein 1 (SREBP-1c).^173^ The fatty acid desaturase 2 (FADS2) gene encodes delta-6 desaturase, and one study has confirmed that NASH is positively related to the expression of the FADS2 in the liver.^174^ To better understand the exact mechanism, Walle et al.^175^ explored the DNA methylation levels of FADS2 from liver biopsy samples of 95 patients with obesity by Infinium HumanMethylation450 BeadChip. They revealed a negative correlation between DNA methylation levels of cg06781209 and cg07999042 and hepatic FADS2 mRNA expression. The results indicated that by modifying DNA methylation, FADS2 mutation participates in the development of NAFLD.^175^ By analysing liver biopsies from 47 patients with NAFLD and 18 control participants, the authors found significantly lower global DNA methylation levels in the liver of patients with NAFLD. In addition, there was a negative correlation between global DNA methylation levels in the liver and hepatic inflammation grade and disease progression. Furthermore, they found a significantly higher serum homocysteine concentration in patients with NAFLD than in the control group, which meant a reduction in SAM. Moreover, a positive correlation was presented between the serum homocysteine concentration and the hepatic steatosis grade and disease progression.^176^

In conclusion, altered DNA methylation levels play a role in the pathogenesis and development of NAFLD. This process is associated with diet and offers a novel idea for improving the prognosis and treatment of patients with NAFLD.

#### Osteoporosis

Some researches have concentrated on the association between osteoporosis and systemic (whole blood) DNA methylation. Cheishvili et al.^177^ explored the DNA methylation signatures in whole blood samples of patients with postmenopausal osteoporosis (PMOP) from the Canadian Multicenter Osteoporosis Study (CaMos) cohort. They found 77 significantly differentially methylated CpG sites, and among them, only five genes may function in bone biology, including actin binding LIM protein family member 2 (ABLIM2), cyclin-dependent kinase-like 5 (CDKL5), Ras homolog family member J (RHOJ), programmed cell death 1 (PDCD1), and zinc finger protein 267 (ZNF267). ABLIM2, CDKL5, RHOJ, and PDCD1 displayed hypermethylation, while ZNF267 showed hypomethylation in patients with osteoporosis.^177^ Whole blood analysis in individuals of Asian Indian origin was performed to analyse CpG methylation in the bone morphogenetic protein 2 (BMP2) promoter through bisulfite-specific polymerase chain reaction (PCR) on the genomic DNA (gDNA) samples. The authors reported a disproportionate allele frequency of methylated ‘C’ between osteoporotic and healthy individuals at the -267 position from the TSS and indicated that BMP2 is hypermethylated in patients with osteoporosis.^178^ However, Fernandez-Rebollo et al.^179^ explored genome-wide DNA methylation profiles of peripheral blood from patients with primary osteoporosis and controls. The results suggested that primary osteoporosis is not affected by disease-specific DNA methylation in peripheral blood. There is inconsistency in the results from different studies, so more research is required on the correlation between osteoporosis and DNA methylation in peripheral blood to understand the mechanisms.

Several researches analysed DNA methylation in bone tissue in patients with osteoporosis. The receptor activator of NF-κB-ligand (RANKL)–the receptor activator of NF-κB (RANK)–the soluble decoy receptor osteoprotegerin (OPG) axis is pivotal for the differentiation and activation of osteoclast.^180^ Wang et al.^181^ explored the implication of DNA methylation on the expression of OPG/RANKL and found that in the osteoporotic fracture (OPF) group, the RANKL gene promoter showed hypermethylation and the OPG gene promoter showed greater methylation. Secreted by osteocytes, sclerostin (SOST) negatively regulates the activity of osteoblasts and osteoclasts on bone surfaces by suppressing the WNT pathway.^182^ Reppe et al.^183^ found that patients with PMOP had elevated SOST promoter methylation, which may decrease suppression of the WNT pathway and promote bone formation. Increased SOST promoter methylation in patients with PMOP was also found in another study. Chromatin immunoprecipitation analysis revealed that increased SOST promoter methylation leads to impairment of the transactivation function of osterix (SP7), runt-related transcription factor 2 (RUNX2), and oestrogen receptor α (ERα).^184^ In addition, bisulfite sequencing revealed that both the OPF group and the non-OPF group presented hypermethylation in SOST gene promoter, while the SOST gene promoter was slightly demethylated in the OPF group.^185^

### The role of histone modification in metabolic diseases

The role of histone modification in metabolic diseases has attracted great interest and there have been tremendous advances in this field. Various histone modifications are involved in the pathogenesis of metabolic diseases through multiple mechanisms.

#### The role of histone acetylation in metabolic disease

##### Diabetes mellitus and its complications

Studies have revealed the role of histone acetylation in diabetes mellitus and its complications. HDAC3 could interact with miR-296-5p to elevate the expression of Bcl-xl, resulting in the enhancement of the anti-apoptotic capacity in lymphocytes and thereby exacerbating type 1 diabetes (T1D).^186^ As HATs and transcriptional co-activators, CBP and its paralogue p300 play critical roles in the β cell identity and functional maturity. By acetylating H3K27 and transcription factors, including FOXO1 and Hnf1α, CBP and p300 are responsible for T2D by regulating transcription.^187^

HDAC5 is significantly increased in renal glomeruli and tubular cells of diabetic mice, which is participated in the high glucose-induced epithelial–mesenchymal transition (EMT) of renal tubular cells. Moreover, methyltransferase‑like 14 (METTL14) could stimulate the expression of PTEN to inactivate the phosphoinositide 3-kinase (PI3K)/AKT signalling pathway, resulting in the downregulation of HDAC5, thus regulating the EMT of renal tubular cells in patients with DKD.^188^ Du et al.^189^ explored the mechanisms of autophagy suppression in Schwann cells in diabetic peripheral neuropathy (DPN). They suggested that under the influence of hyperglycaemia, HDAC1 interacts with Atg3 to downregulate autophagy markers, such as LC3-I and LC3-II. The Janus kinase (JAK)–signal transducer and activator of transcription 3 (STAT3) signalling pathway is activated by hyperglycaemia, and STAT3 phosphorylation enhances HDAC1 and downregulates autophagy markers, including P62.^189^ HDAC3 is highly expressed in the retina tissues of DR mice. By interacting with miR-296-5p, HDAC3 upregulated the expression of GNAI2 in retina tissues, which promoted apoptosis of retinal ganglion cells in DR models.^190^ Macrophages are vital for the process of diabetic wound healing. Males absent on the first (MOF), a HAT, serves as a coactivator of tumor necrosis factor-alpha (TNF-α)/ NF-κB signalling. In 2020, MOF was reported to inhibit diabetic wound healing by increasing the expression of inflammatory genes associated with NF-κB via promoting acetylation of H4K16 in wound macrophages.^191^

##### Obesity

Increasing evidence suggests that histone acetylation is related to obesity. MOF is one of the lysine acetyltransferases (KATs), which are involved in the acetylation of histone H4 at lysine 16 (H4K16ac). H4K16ac induced by MOF acts as a regulator to maintain glucose uptake and lipid storage in adipocytes by interacting with peroxisome proliferator-activated receptor gamma (PPARγ), thereby exacerbating the progress of obesity.^192^ HDAC3 affects the differentiation of adipocytes by modulating adipocyte phenotype. HDAC3 knockdown could not only regulate adipocyte pro-inflammatory profile, but also promote the expression of transcriptional regulators related to adipogenesis, including Cebpb, Cebpa, Srebf1c, and PPARγ.^193^ There is low HDAC6 expression in adipose tissues of humans with obesity and animal models of obesity. HDAC6 could regulate lipid storage by acetylating cell death-inducing DFFA-like effector C (CIDEC), a lipid droplet-binding protein.^194^ Of interest, Lieber et al.^195^ observed increased weight gain in HDAC6-deficient male mice. Further study indicated that loss of HDAC6 changes the gut microbiota composition, with increased *Bacteroides* and *Parabacteroides* and decreased S24-7 family and *Lactobacillus*; these changes may aggravate obesity by inhibiting the capacity of regulatory T cells (Tregs).^195^ HDAC11 is also involved in obesity and obesity-related disease.^196^ HDAC11 knockdown effectively alleviates obesity-related disease by restraining hypercholesterolemia, liver steatosis, and damage, and by increasing insulin sensitivity and glucose tolerance. Exploration of the underlying mechanisms indicated that loss of HDAC11 stimulates the expression of UCP1 in brown adipose tissue (BAT) and increases the thermogenic capacity. Besides, oxygen consumption and metabolic activity are enhanced by HDAC11 deficiency, and carnitine palmitoyltransferase 1 (CPT1), an important enzyme for regulating mitochondrial long-chain fatty acid β-oxidation (FAO), is increased in HDAC11 knockdown mice. Furthermore, deletion of HDAC11 promotes the adiponectin–adipoR–5’ AMP-activated protein kinase (AMPK) signalling pathway by increasing the adiponectin levels in the liver.^196^ In the same year, the same research team found that loss of HDAC11 promotes the formation of BAT and beiging of white adipose tissue (WAT). By binding to BRD2, HDAC11 inhibits the BAT transcriptional programme to suppress the thermogenic potential of adipose tissue, contributing to obesity.^197^

##### NAFLD

According to recent studies, histone acetylation is related to NAFLD. Lactate accumulation in the liver could accelerate the pathogenesis of NASH. Acetylation of lactate dehydrogenase B (LDHB) K82 mediated by PCAF induces the accumulation of lactate by suppressing the LDHB activity and inhibiting lactate clearance, which aggravates inflammatory responses and lipid deposition in the liver.^198^ Besides, H3K27 acetylation at the promoter of lncRNA NEAT1 facilitates its transcription and exacerbates the development of NAFLD by accelerating lipid accumulation in the liver through sponging miR-212-5p and enhancing the expression of GRIA3.^199^ Zhou et al.^200^ suggested that nuclear receptor subfamily 2, group F, member 6 (NR2F6) plays a critical role in the pathogenesis of NAFLD. Expressed highly in patients with NAFLD, NR2F6 interacts with and upregulates the fatty acid (FA) translocase CD36 in hepatocytes, and then facilitates histone acetylation at the promoter of nuclear receptor coactivator 1, leading to elevated hepatic TG. Metformin can reverse this effect and might serve as a potential treatment strategy.^200^ In addition, decreased production of reactive oxygen species (ROS) induced by the loss of CD36 aggravates the pathogenesis of NASH by upregulating monocyte chemotactic protein-1 (MCP-1) in hepatocytes, which accelerates the inflammatory response and fibrosis in the liver by facilitating macrophage migration to the liver. HDAC2 could suppress transcriptional activation of MCP-1 by inhibiting acetyl H3. However, HDAC2 is reduced in CD36 deficiency mice due to the decreased ROS production, thus aggravating NASH.^201^ S100 calcium binding protein A11 (S100A11) is induced by an HFD. Acting as a deacetylase of FOXO1, HDAC6 is downregulated by binding to S100A11, which increases the acetylation and activity of FOXO1, leading to lipogenesis and activation of autophagy in the liver, thus exacerbating liver steatosis.^202^

##### Osteoporosis

Histone acetylation has been implicated in the development of osteoporosis. The zinc-finger transcription factor ZEB1 is expressed at a low level in the skeletal endothelium of patients with osteoporosis and mouse models of that disease. ZEB1 deficiency decreases histone acetylation on Notch1 promoters and inhibits the Notch signalling pathway, which is related to osteogenesis.^203^ In addition, histone acetylation is involved in ameliorating osteoporosis via miR-29a. The underlying mechanism is that miR-29a inhibits H3K27ac at CXCL12 promoters mediated by the histone acetyltransferase PCAF, thus downregulating CXCL12 and suppressing osteoclast differentiation.^204^ In addition, PCAF could facilitate osteogenic differentiation of mesenchymal stem cells (MSCs) via BMP signalling pathway by promoting H3K9 acetylation.^205^

RUNX2 acts as an important regulator for the osteogenic differentiation potential of bone marrow mesenchymal stem cells (BMSCs). HDAC6 and androgen receptor (AR) interact with the RUNX2 promoter competitively to regulate the expression of RUNX2 in BMSCs. HDAC6 accumulation in the RUNX2 promoter would deacetylate it and decrease the expression of RUNX2, contributing to age-related bone loss.^206^ Nucleosome assembly protein 1-like 2 (NAP1L2) restrains osteogenic differentiation of BMSCs. Acting as a histone chaperone, NAP1L2 inhibits acetylation of lysine 14 in histone 3 (H3K14ac) on promoters of osteogenic genes, including RUNX2 and SP7, by recruiting SIRT1, a Class III HDAC.^207^ Mechanical stimulation accelerates the osteogenic differentiation of BMSCs by downregulating HDAC1. Wang et al.^208^ revealed that HDAC1 could suppress the transcription of jagged 1 (JAG1), an important regulator of osteogenesis, and inhibit the Notch signalling pathway mediated by JAG1. In addition, general control nonderepressible 5 (GCN5), a HAT, suppresses the osteogenic differentiation of MSCs through preventing NF-κB transcription and blocking the NF-κB signalling pathway.^209^

#### The role of other histone modifications in metabolic disease

Other histone modifications, including methylation, demethylation, phosphorylation, ubiquitination, and butyrylation, are involved in the pathogenesis of metabolic disease. Kimball et al.^210^ found that methyltransferase Setdb2 is beneficial for wound healing. By trimethylating lysine 9 on histone 3 (H3K9me3) at different gene promoters, Setdb2 not only regulates macrophage polarity by inhibiting the transcription of inflammatory cytokine genes, including interleukin 1 beta (IL-1β), nitric oxide synthase 2 (NOS2), and TNFα, but also decreases uric acid (UA) production by restraining the activity of xanthine oxo-reductase (XOR). However, with interferon beta (IFNβ) modulation Setdb2 expression in wound macrophages is decreased under diabetic conditions, thereby resulting in a persistent inflammatory phenotype of macrophage in diabetic wounds.^210^ In addition, DOT1L, an HMT, alleviates osteoporosis by suppressing osteoclastogenesis. DOT1L interference enhances the expression of CD9 and matrix metallopeptidase 9 (MMP9), proteins associated with osteoclast fusion and resorption, but also promotes cell migration, autophagy activity, and ROS production in pre-osteoclasts.^211^ It is reported that the HDM plant homeodomain finger 2 (Phf2) could retard the progression of NAFLD by promoting H3K9me2 demethylation at specific gene promoters. Acting as a transcriptional co-activator of carbohydrate-responsive element binding protein (ChREBP), Phf2 enhances the expression of stearoyl-CoA desaturase 1(SCD1) and promotes the conversion of saturated fatty acids (SFA) into mono-unsaturated fatty acids (MUFA), reducing insulin resistance and hepatic inflammation. Furthermore, Phf2 could protect the liver against oxidative stress by activating Nrf2.^212^

A few studies have focused on histone phosphorylation, ubiquitination, butyrylation, histone ADP-ribosylation, and histone crotonylation in metabolic diseases. Alghamdi and colleagues^213^ found elevated phosphorylation of histone H3 on serine residue 10 (phospho-histone H3Ser10) in the glomeruli of patients with diabetic kidney disease. They indicated that increased glomerular endothelial vascular cell adhesion protein 1 (VCAM-1) is induced by CCL2/CCR2 signalling via phosphorylation of H3Ser10 at the promoter of VCAM-1. Besides, inhibition of mitogen- and stress-activated protein kinases 1/2 (MSK1/2) would decrease the level of H3Ser10 phosphorylation.^213^ Histone 2B ubiquitin ligase RNF40 is critical for bone formation and remodelling. By modulating the expression of RANKL, RNF40 promotes osteoblast differentiation in early stages.^214^ Kbhb acts as a novel histone mark. Further study revealed that elevated Kbhb on histone H3 lysine 9 (H3K9bhb) induced by starvation is related to diabetes through the PPAR signalling pathway.^103^ By transferring ADP-ribose to target proteins via using NAD + as substrate, Poly (ADP-Ribose) polymerases (PARPs) are participated in several biological processes. By ADP-ribosylating histone H2B at serine 7 of the NFATc1 promoter, PARP1 downregulated the expression of NFATc1, which is crucial for the macrophage differentiation into osteoclasts.^215^ A recent study reported that in patients with T2D, histone H3K27 crotonylation in the GLUT4 promoter region was regulated by lncRNA EPB41L4A-AS1 /GCN5 complex, which decreased the expression levels of GLUT4 and prevented glucose uptake by muscle cells.^216^

### The role of chromatin remodelling in metabolic disease

#### Diabetes mellitus and its complications

BRD7 and BRD9 can recognise acetylated lysine.^217^ BRD7 is a component of polybromo-associated BRG1-associated factor (PBAF)-specific SWI/SNF, while BRD9 belongs to the BAF complex.^218,219^ In 2018, Wei et al.^220^ explored the role of vitamin D receptor (VDR) in T2D and found that the balance between PBAF-BRD7 and BAF-BRD9 is important for the VDR-induced pro-survival and anti-inflammatory response. Moreover, BRD9 alleviates hyperglycaemia by promoting VDR association with PBAF to change chromatin accessibility, thus restoring β cell function.^220^ Pdx1 is a diabetes-linked transcription factor, and SWI/SNF is essential for Pdx1 to interact with the Ins gene enhancer and then regulate the function of mature islet β cell and pancreatic progenitor cell proliferation.^221^ As a linker between transcription factors and the SWI/SNF core complex, BAF subunits, including BAF60a, BAF60b, and BAF60c, play a vital important role in diabetes. Recently, researchers found that BAF60a is participated in the pathogenesis of T2D. Kong et al.^222^ proved that BAF60a interacts with the transcription factor Atf3 to regulate adipose tissue macrophages (ATMs) inflammation activation and insulin resistance in WAT through chromatin remodelling–mediated epigenetic mechanisms.^222^ BAF60c, also called Smarcd3, is a transcriptional cofactor enriched in fast-twitch muscles. One study suggested that transgenic expression of BAF60c can activate the glycolytic pathway in muscles to protect mice from diet-induced insulin resistance.^223^ The CHD family of remodellers is also associated with diabetes. CHD4 interacts with transcription factor 19 (TCF19), which is involved in the maintenance of pancreatic β cells via regulation of cell proliferation and apoptosis.^224^

#### NAFLD

The BAF subunit is also vital in NAFLD. Li et al.^225^ found that by interacting with PPARα and PGC-1α, BAF60a induces the transcriptional activation of peroxisomal and mitochondrial fat-oxidation genes and regulates hepatic FAO. In addition, BAF60a acts as a diet-sensitive subunit and promotes the expression of genes related to hepatic bile acid metabolism and cholesterol absorption.^226^ Wang et al.^227^ found that BAF60c is an important chromatin remodelling component for lipogenic gene transcription in the liver, which interacts with upstream stimulating factor-1 (USF-1), leading to USF-1 phosphorylation by DNA-PK and acetylation by PCAF.

#### Osteoporosis

One study suggested that INO80 is essential for osteogenic differentiation of human bone marrow–derived human mesenchymal stem cells (hBMSCs), which interact with Wdr5 in MSC and positively regulate the WNT signalling transduction, contributing to changes in the expression of osteoblast-specific genes, including RUNX2, Col1α1, Osx, and OCNb.^228^ SWI/SNF controls lineage selection in MSCs – for example, SWI/SNF can redirect the adipogenic potential of bone marrow–derived MSCs to osteoblasts, which may provide a new treatment to protect against age-related osteoporosis.^229^ In addition, expression of nuclear receptor binding SET domain protein 2 (NSD2) was induced by melatonin, which may prevent ageing-associated bone loss by the rebalancing of H3K27me3 and H3K36me2 modifications to remodel chromatin of the osteogenic genes.^230^

### The role of ncRNA regulation in metabolic disease

There is an abundance of research on the relationship between ncRNAs, including miRNA, lncRNA, and circRNA, and metabolic disease. We summarise this content separately in Table 2 (miRNA), Table 3 (lncRNA), and Table 4 (circRNA).

**Table 2:** Regulation of miRNA in metabolic disease

Diseases	Major regulator	Target gene	Effect	References
T1D	miR-150	PUMA	↓	^231^
T2D	miR-200c	ETV5	↑	^232^
GDM	miR-423-5p	IGF1R/GYS1	↑	^234^
GDM	miR-122-5p	G6PC3/FDFT1	↓	^234^
GDM	miR-199a-5p	Trpc3, MeCP2	↑	^235^
DKD	miR-146a-5p	TRAF6, STAT1	↓	^236^
diabetic wound healing	miR-129	TRAF6	↑	^237^
diabetic vascular damage	miR-142-5p	IL-1β	↑	^238^
Obesity	miR-342-3p	Snap25	↑	^239^
Obesity	miR-7, miR-17-92	FOXO1	↑	^240^
Obesity	miR-155	PPARG, GLUT4	↓	^241^
Obesity	miR-690	Nadk	↓	^242^
Obesity	miR-34a	KLF4	↑	^243^
Obesity	miR-122	VDR, SREBF1	↑	^244^
NAFLD	miR-122	Sirt1	↑	^246^
NAFLD	miR-20b	PPARA	↑	^247^
NAFLD	miR-223	TAZ	↓	^248^
NAFLD	miR-378	NF-κB	↑	^249^
NAFLD	miR-214-3p	Ulk1	↑	^250^
NAFLD	miR-26a	eukaryotic initiation factor 2α	↓	^251^
Osteoporosis	miR-100	AKT	↓	^252^
Osteoporosis	miR-152-5p	ATG14	↓	^253^
Osteoporosis	miR-34a-5p	TGF-β-induced factor homeobox 2, Notch1	↓	^254^
Osteoporosis	miR-214-3p	tensin homolog, transcription factor 4	↑	^254^
Osteoporosis	miR-1224-5p	ADCY2	↓	^255^
Osteoporosis	miR-26a-5p	HDAC4	↓	^256^
Osteoporosis	miR-150	FNDC5, irisin	↑	^257^
Osteoporosis	miR-21-5p	KLF3	↓	^258^
Osteoporosis	miR-214-5p	ITGA7	↑	^259^

**Table 3:** Regulation of lncRNA in metabolic disease

Diseases	Major regulator	Target gene	Effect	References
T1D	lncRNA SRAs	miR-146b	↑	^260^
T2D	lncRNA MALAT1	Nrf2, JNK, Akt, IRS-1	↓	^261^
GDM	lncRNA HOTTIP	WNT7A	↓	^262^
DKD	lncRNA MALAT1	LIN28A	↑	^263^
DR	lncRNA ZNF503-AS1	TGF-β	↑	^264^
diabetic wound healing	lncH19	p53, GDF15	↑	^265^
Obesity	lncRNA RP11-142A22.4	miR-587	↑	^266^
Obesity	lnc13728	ZBED3	↑	^267^
Obesity	lncH19	miR-30a	↑	^268^
Obesity	lncFR332443	RUNX1, MAPK	↓	^269^
Obesity	lncRNA MIR99AHG	miR-29b-3p	↑	^270^
Obesity	lncRNA U90926	PPARγ, PPARγ2	↓	^271^
Obesity	lncRNA XIST	C/EBPα	↓	^272^
Obesity	FOXC2-AS1	UCP1	↓	^273^
NAFLD	LncRNA Gm15622	miR-742-3p	↑	^274^
NAFLD	Hilnc	IGF2BP2	↑	^275^
NAFLD	lncRNA MALAT1	CXCL5	↑	^276^
NAFLD	lncRNA NEAT1	miR-122	↑	^277^
NAFLD	lncRNA HULC	MAPK	↑	^278^
NAFLD	lncRNA-Gm9795	TNF, IL-6, and IL-1	↑	^279^
NAFLD	lncRNA Platr4	NF-κB	↓	^280^
Osteoporosis	lncRNA MIAT	miR-150-5p	↑	^281^
Osteoporosis	lncRNA RAD51-AS1	YBX1	↓	^282^
Osteoporosis	lncRNA TCONS_00072128	caspase 8	↑	^283^
Osteoporosis	lncRNA TUG1	Hippo	↓	^284^
Osteoporosis	LncDIF	miR-489-3p	↑	^285^
Osteoporosis	LncNEAT1	Smurf1	↓	^286^
Osteoporosis	lncRNA LIOCE	Osterix	↓	^287^
Osteoporosis	lncRNA NRON	NFATc1	↓	^288^
Osteoporosis	lncAK077216	NIP45	↑	^289^

**Table 4:** Regulation of circRNA in metabolic disease

Diseases	Major regulator	Target gene	Effect	References
T2D	circ_0071336	miR-93-5p	↑	^290^
T1D	circPPM1F	HuR, FUS, EIF4A3	↑	^291^
GDM	circMAP3K4	miR-6795-5p	↑	^292^
DR	circNNT	miR-320b	↓	^293^
DC	circHIPK3	PTEN	↓	^294^
diabetic wounds healing	circARHGAP12	miR-301b-3p	↑	^295^
Obesity	circSAMD4A	miR-138-5p	↓	^296^
Obesity	circFLT1	miR-93	↓	^297^
Obesity	circPPARγ	miR-92a-3p	↓	^298^
Obesity	circFUT10	let-7c	↑	^299^
Obesity	circOgdh	miR-34a-5p	↓	^300^
Obesity	circARF3	miR-103	↓	^302^
NAFLD	circRNA_0001805	miR-106a-5p, miR-320a	↓	^303^
NAFLD	circ_0057558	miR-206	↑	^304^
NAFLD	circ_0048179	miR-188-3p	↓	^305^
NAFLD	circRNA SCAR	PGC-1α	↓	^306^
NAFLD	circRNA_002581	miR-122	↓	^307^
Osteoporosis	circIGSF11	miR-199b-5p	↓	^308^
Osteoporosis	circ_0074834	miR-942-5p	↓	^309^
Osteoporosis	circRNA_0016624	miR-98	↓	^310^
Osteoporosis	circRNA_0048211	miR-93-5p	↓	^311^
Osteoporosis	circStag1	HuR	↓	^312^
Osteoporosis	circRNA AFF4	miR-7223-5p	↓	^313^
Osteoporosis	circHIPK3	miR-124	↓	^314^
Osteoporosis	circRNA_28313	miR-195a	↑	^315^
Osteoporosis	circBBS9	miR-423-3p	↓	^316^

#### The role of miRNAs in metabolic disease

##### Diabetes mellitus and its complications

miRNAs are involved in the pathogenesis of both T1D and T2D. NF-κB prevents the occurrence of T1D by increasing the expression of miR-150, which downregulates the expression of p53 upregulated modulator of apoptosis (PUMA) to suppress T1D-induced inflammation and β cell apoptosis.^231^ miR-200c presents higher expression in islets from patients with T2D. Further research indicated that miR-200c decreases the secretion of insulin by targeting transcription factor ETV5.^232^

Gestational diabetes mellitus (GDM) is a special type of diabetes mellitus in a mother develops hyperglycaemia during pregnancy that is ameliorated after she gives birth.^233^ Ye and colleagues^234^ explored the miRNA expression profile of plasma exosomes in women with GDM. By using high-throughput small RNA sequencing in 12 pregnant women with normal glucose tolerance (NGT) and 12 women with GDM, they identified 22 differentially expressed exosomal miRNAs and verified five of them by real-time reverse transcription-PCR (qRT-PCR), including upregulated miR-423-5p, and downregulated miR-99a-5p, miR-122-5p, miR-148a-3p, and miR-192-5p. miR-423-5p and miR-122-5p are participated in the regulation of metabolism in GDM by targeting IGF1R/GYS1 and G6PC3/FDFT1; these effects are related to AMPK signalling pathways.^234^ In addition, miR-199a-5p is increased in the placenta and placental villi of women with GDM compared with normal pregnant women. miR-199a-5p can reduce the expression of canonical transient receptor potential 3 (Trpc3) and methyl CpG-binding protein 2 (MeCP2) to modulate methylation levels and the glucose pathway.^235^

Diabetes can affect several organs and cause diverse complications. miRNAs are participated in the complications, such as DKD, diabetic wounds, and diabetic vascular damage. Zhang et al.^236^ revealed that exosomal miR-146a-5p from the human umbilical cord–derived MSCs (UC-MSCs) protects against DKD in rats through targeting tumor necrosis factor receptor-associated factor-6 (TRAF6) and STAT1 to induce M2 macrophage polarisation.^236^ Resveratrol promotes diabetic wound healing. Hu et al.^237^ explored the molecular mechanism of resveratrol in diabetic wound healing and found that it promotes the transportation of extracellular vesicles (EVs) containing miR-129derived from MSCs. By binding to TRAF6, miR-129 improves the proliferative, migratory, and tube formation potentials of human umbilical vein endothelial cells (HUVECs), thus contributing to diabetic wound healing in T1D. A recent study revealed that exosomes derived from high glucose–induced monocytes cause vascular damage by reducing migration and increasing ROS production in HUVECs. Moreover, researchers revealed that exosomal miR-142-5p is participated in the pathogenesis of vascular damage by targeting IL-1β.^238^

##### Obesity

miRNA can be a vital regulator for the pathogenesis of obesity. Zhang et al.^239^ performed RNA sequencing and found that miR-342-3p and its host gene Evl, which are co-expressed in the hypothalamic arcuate nucleus neurons, are increased in the brain and adipose tissues of mice with diet-induced obesity. By targeting Snap25, miR-342-3p overexpression regulates NPYpSTAT3 and POMCpSTAT3 neurons, thereby leading to functional impairment in hypothalamic neurons and excess food intake.^239^ Similarly, miR-7 and miR-17–92, which are expressed in proopiomelanocortin (POMC)-expressing neurons in the arcuate nucleus (ARC) of the hypothalamus, are partially responsible for diet-induced obesity. Moreover, body weight regulation mediated by miR-7 and miR-17–92 present sexual dimorphism, and altered expression of genes differentially expressed in the sexes in the ARC, such as FOXO1, may contribute to the characteristics.^240^

As a common content of exosomes, exosomal miRNA derived from different cells and tissues are revealed to be participated in the pathogenesis of obesity. miR-155 expression is elevated 6.7-fold in ATMs of people with obesity. Exosomal miR-155 derived from ATM could be absorbed by neighbouring adipocytes and regulate adipocyte metabolism by targeting PPARγ and GLUT4.^241^ M2 polarised bone marrow–derived macrophages (BMDMs) secrete exosomes containing miR-690, which improves insulin sensitivity and glucose tolerance in obese mice by targeting Nadk.^242^ In addition, Pan et al.^243^ found upregulated miR-34a expression in adipose tissues of obese mice. miR-34a derived from adipocyte exosomes is delivered to macrophages and inhibits the expression of Krüppel-like factor 4 (KLF4) to prevent M2 polarisation, thus aggravating metabolic inflammation and insulin resistance induced by obesity.^243^ Of interest, exosomes from adipose tissue could promote adipogenesis and exacerbate obesity. miR-122 is enriched in adipose tissue–derived exosomes; it can target VDR and interact with the BS1 region of the sterol regulatory element-binding transcription factor 1 (SREBF1) promoter to suppress VDR and SREBF1 expression, thus leading to the pathogenesis of obesity.^244^

In general, miRNAs, including exosomal miRNA, are participated in the development of obesity by regulating the expression of genes related to obesity, which will offer new insights into the prognoses and treatment for obesity.

##### NAFLD

Several miRNAs regulate hepatic lipid metabolism. miR-122 accounts for nearly 70% of all miRNA expressed in the liver.^245^ miR-122 is upregulated in hepatocytes of patients with NAFLD and plays a pivotal role in the pathogenesis of NAFLD. miR-122 aggravates hepatic lipogenesis by targeting SIRT1 and activating the LKB1-AMPK cascade.^246^ Lee et al.^247^ revealed that hepatic miR-20b exacerbates the development of NAFLD by suppressing PPARα, which prevents mitochondrial biogenesis and FAO. miRNAs have also been implicated in NAFLD-associated fibrosis. Many cytokines, particularly IL-6, play critical roles in NAFLD. A recent study suggested that myeloid cell–specific IL-6 increases the expression of exosome biogenesis-related genes and facilitated the production of miR-223-enriched exosomes derived from macrophages. Exosomes containing miR-223 are transported to hepatocytes and miR-223 suppresses the expression of transcriptional activator with PDZ-binding motif (TAZ), which has been acknowledged to exacerbate NASH fibrosis.^248^ Besides, by regulating the NF-κB–TNFα signalling pathway, miR-378 aggravates inflammation and fibrosis in the liver.^249^

Studies have shown that autophagy is involved in NAFLD. In both patients and mice with fatty liver, there is decreased expression of Ulk1, an autophagy-related gene. Further research found that in hepatocytes, miR-214–3p decreased the expression of Ulk1, thus inhibiting autophagic activity to promote fatty liver disease.^250^ Moreover, stress-activated pathways are participated in the pathogenesis of NAFLD. miR-26a expression is induced by endoplasmic reticulum (ER) stress in liver cells and is reported to decrease in the liver of patients with NAFLD. Furthermore, miR-26a downregulates the expression of the eukaryotic initiation factor 2α, which acts as an important ER stress effector and regulates cellular translation. ER stress–evoked miR-26a upregulation could offer a novel therapeutic strategy for NAFLD.^251^

In general, miRNA is widely involved in the development of NAFLD by multiple mechanisms, including regulating hepatic lipid metabolism, autophagy, and ER stress.

##### Osteoporosis

Several stem cells have the potential for osteogenic differentiation, such as BMSCs and mesenchymal stem cells from the mandible (MMSCs-M). Researchers found significantly increased miR-100 expression in bone tissues and BMSCs of osteoporotic mice. Moreover, miR-100 reduction accelerates bone regeneration defects of BMSCs in osteoporotic mice by the AKT–mammalian target of rapamycin (mTOR) pathway.^252^ Li et al.^253^ revealed that miR-152-5p is increased in MMSCs-M, and they observed autophagy-related genes, proteins, and autophagosomes in the ovariectomy (OVX) group. They found that downregulating miR-152-5p facilitates the osteogenic differentiation of MMSCs-M through accelerating autophagy-related protein homologue 14 (ATG14)-mediated autophagy with reduced accumulation of endogenous ROS.^253^

The balance between osteoblasts and osteoclasts is crucial for bone metabolism. A recent study indicated that delivering recombinant adeno-associated viral (rAAV) vectors to bone and modulating the expression of miR-214-3p and miR-34a-5p can influence both osteoblasts and osteoclasts to treat osteoporosis. Increasing the expression of miR-34a-5p could decrease TGF-β-induced factor homeobox 2 in osteoclasts and Notch1 in osteoblasts, while downregulating miR-214-3p elevates tensin homologue in osteoclasts and activates transcription factor 4 in osteoblasts.^254^ miR-1224-5p is a vital bone osteogenic regulator and alleviates osteoporosis by targeting ADCY2 to promote osteoblast differentiation through the Rap1 signalling pathway and inhibiting RANKL-induced osteoclast differentiation.^255^ miR-26a-5p is enriched in urine-derived stem cell extracellular vesicles (USCs-EVs).^256^ By inhibiting HDAC4, miR-26a-5p derived from USCs-EVs can activate the HIF-1α and vascular endothelial growth factor A (VEGFA) pathway to facilitate the differentiation of osteogenic precursor cells, thus alleviating diabetic osteoporosis (DOP).^256^ miR-150 expression is elevated in patients with diabetes via oxidative stress and by interacting with 3’-UTR of FNDC5 and irisin. miR-150 downregulates FNDC5 and irisin expression, which induces pyroptosis in diabetic bone tissue. Irisin, induced by physical exercise or administered directly, can help to reverse FNDC5 and irisin downregulation to facilitate osteoblast function and bone formation.^257^ Exosomal miR-21-5p from BMSCs could promote osteoblastic differentiation and ameliorate osteoporosis by interacting with KLF3.^258^ In osteoclasts, activating transcription factor 1 (ATF1) can activate miR-214-5p transcriptionally and reduce the expression of ITGA7, thus contributing to osteoclastogenesis and altering OVX-induced bone absorption.^259^

#### The role of lncRNAs in metabolic disease

##### Diabetes mellitus and its complications

lncRNAs are implicated in various types of diabetes mellitus. lncRNA steroid receptor RNA activators (SRAs) are highly expressed in peripheral blood mononuclear cells (PBMCs) and plasma samples from patients with T1D. lncRNA SRAs regulate the functional genes of Tregs and inhibit the expression of miR-146b in β cells to activate the interleukin-1 receptor-associated kinase 1 (IRAK1)–lactate dehydrogenase A (LDHA)–phosphorylated LDHA (pLDHA) signalling pathway, which induces apoptosis of β cells and facilitates T1D pathogenesis.^260^ Reduction of the lncRNA metastasis-associated lung adenocarcinoma transcript 1 (MALAT1) was reported to decrease ROS by targeting Nrf2. Besides, downregulated MALAT1 inhibits JNK activity, AKT phosphorylation, and insulin receptor substrate 1 (IRS1) activation induced by insulin, thereby regulating the sensitivity to insulin in T2D.^261^ Cao et al.^262^ observed increased miR-423-5p and decreased lncRNA HOTTIP and wingless-type MMTV integration site family member 7 A (WNT7A) in GDM mice. By sponging miR-423-5p, HOTTIP elevates the levels of WNT7A to relieve hepatic gluconeogenesis and insulin resistance in GDM mice.^262^

lncRNAs also function in the complications of diabetes mellitus, such as DKD, DR, and diabetic wound healing. MALAT1 are involved in DKD. It not only interacts with LIN28A directly, but also promotes the interaction between LIN28A and Nox4 to activate the AMPK–mTOR signalling pathway and to increase the stability of Nox4. These actions would allow MALAT1 to exacerbate high glucose–induced renal tubular epithelial injury.^263^ ZNF503-AS1 is a novel lncRNA with higher expression in patients with DR than in control patients. By activating TGF-β signalling, ZNF503-AS1 overexpression promotes apoptosis and suppresses proliferation.^264^ Yu et al.^265^ found that there is low expression of the lncH19 in the wound-healing cutaneous tissue of patients and mice with T2D; this state can facilitate macrophage infiltration as well as dermal fibroblast proliferation in injured skin by suppressing the activity of p53 and GDF15 releasement. Besides, exosomes derived from adipocyte progenitor cells deliver lncH19 to injured tissue, thereby accelerating diabetic wound healing.^265^

##### Obesity

lncRNAs are participated in the pathogenesis of obesity by regulating adipogenesis and promoting adipocyte differentiation. The lncRNA RP11-142A22.4 is increased in visceral adipose tissue. A study suggested that RP11-142A22.4 interacts with miR-587 and then regulates the expression of WNT5β, thus contributing to adipogenesis.^266^ After human adipose-derived MSCs (hADSCs) adipogenic differentiation, there is significantly increased lnc13728 expression that is positively correlated with adipogenesis-related gene expression. lnc13728 facilitates adipogenic differentiation in hADSCs by inhibiting the WNT–β-catenin pathway through ZBED3 upregulation.^267^ Similarly, lncH19 promotes hADSC adipogenic differentiation by sponging miR-30a to enhance C8orf4 expression.^268^ On the contrary, lncFR332443 suppresses preadipocyte differentiation by increasing RUNX1 expression and inhibiting the mitogen-activate protein kinase (MAPK)–extracellular signal-regulated kinase 1/2 (ERK1/2) and MAPK–p38 signalling pathways.^269^ Besides, lncRNA MIR99AHG promotes adipocyte differentiation by binding to miR-29b-3p to regulate PPARγ, while lncRNA U90926 inhibits 3T3-L1 adipocyte differentiation by preventing the transactivation of PPARγ or PPARγ2.^270,271^ Based on recent reports, the lncRNA XIST could act as a novel target to treat obesity. XIST expression in adipose tissue is higher in female than in male individuals and XIST expression is increased during brown adipocyte differentiation. Investigation of the underlying mechanisms indicates that XIST resists obesity by activating BAT and by binding to CCAAT enhancer-binding protein α (C/EBPα).^272^ FOXC2-AS1 is a lncRNA that is upregulated in human adipocytes. Nevertheless, FOXC2-AS1 is reduced during white adipocyte differentiation. Further study revealed that FOXC2-AS1 decreases the UCP1 protein level and thermogenic capacity via the autophagy signalling pathway to promote white adipocyte browning, which may provide a novel strategy to treat obesity.^273^

##### NAFLD

lncRNAs are also participated in the regulation of lipid metabolism. The lncRNA Gm15622 is highly expressed in the liver of obese mice. During the exploration of the role of Gm15622 in the development of NAFLD, Ma et al.^274^ found that Gm15622 upregulates the transcriptional regulator SREBP-1c and stimulates hepatic lipid accumulation in the liver by sequestering miR-742-3p.^274^ The Hedgehog signalling pathway has been reported to be responsible for hepatic lipid metabolism. Recently, researchers found that a novel lncRNA, Hedgehog signalling–induced lncRNA (Hilnc), regulates hepatic lipid metabolism by binding to IGF2BP2 to stabilise PPARγ mRNA. This action may facilitate the progress of hepatic steatosis. Moreover, this effect can be reversed by metformin.^275^

By exacerbating the progress of liver fibrosis, MALAT1 plays a crucial role in the pathogenesis of NAFLD. MALAT1 expression is modulated by insulin and hyperglycaemia in HepG2 cells, but only regulated by insulin in hepatic stellate cells. By increasing CXCL5 expression, MALAT1 is responsible for inflammation and fibrosis in NASH.^276^ Besides, expression of the lncRNA NEAT1 is elevated in carbon tetrachloride (CCl4)-induced mouse liver fibrosis models and activated hepatic stellate cells (HSCs).^277^ The underlying mechanism is that NEAT1 increases KLF6 expression by sponging miR-122, thus contributing to the activation of HSCs and facilitating the progress of liver fibrosis. Thereby, NEAT1 inhibition could offer a novel therapy to treat NASH.^277^ In addition, by inhibiting the MAPK signalling pathway, the lncRNA HULC may exacerbate the pathogenesis of NAFLD by promoting the progression of hepatic fibrosis and hepatocyte apoptosis.^278^

lncRNAs are also participated in the pathogenesis of NAFLD by activating the inflammatory response. Ye et al.^279^ found that the lncRNA Gm9795 accelerates the pathogenesis of NAFLD by stimulating the expression of inflammatory mediators in NASH, including TNF, IL-6, and IL-1, instead of increasing fat accumulation. The expression of inflammatory mediators is induced by the elevation of critical molecules in ER stress, which regulate the JNK and NF-κB pathways.^279^ In addition, Platr4, an oscillating and NF-κB-related lncRNA, mitigates NASH by suppressing the NF-κB signalling pathway; this action suppresses transcription of the inflammasome components apoptosis-associated speck-like protein containing a CARD (ASC) and NOD-like receptor family pyrin domain containing 3 (NLRP3).^280^

##### Osteoporosis

BMSCs could be a good choice to treat osteoporosis given their great osteogenic potential, lncRNAs function in BMSC differentiation by acting as miRNA sponges. In patients with osteoporosis, expression of the lncRNA MIAT is increased significantly when miR-150-5p is downregulated, and the serum indicators of osteogenic differentiation are decreased.^281^ In addition, lncRNAs can help regulate BMSCs by binding to other molecules. RAD51-AS1, which is mainly located in the nucleus, presents low expression in BMSCs of patients with osteoporosis. RAD51-AS1 can bind YBX1 and prevent the translation of Smurf2 and SMAD7 and increase the transcription of SIVA1 and PCNA, thus activating the TGF-β signalling pathway and promoting the proliferation, osteogenic differentiation, and ectopic bone formation of BMSCs.^282^ Exosomes have always been a major focus of this research. The lncRNA TCONS_00072128 derived from serum of patients with PMOP downregulates caspase-8 expression and inhibits BMSC osteogenic differentiation.^283^

The role of lncRNAs in osteoblasts is also worth exploring. Recently, Han et al.^284^ revealed that epigallocatechin gallate (EGCG) can ameliorate the suppression of osteoblastic differentiation induced by TNF-α and treat osteoporosis. Specifically, EGCG enhances the expression of the lncRNA TUG1 and prevents the Hippo/YAP signalling pathway.^284^ lncDIF suppresses osteoblast differentiation. It contains several 53 nucleotide repeats at the trailing end, which may sponge miR-489-3p and upregulate the expression of SMAD2, an inhibitor of osteoblast differentiation.^285^ NEAT1 is a mechanosensitive lncRNA that is downregulated under mechanical stimulation, such as simulated microgravity. NEAT1 deficiency in osteoblasts decreases their sensitivity to mechanical stimulation. NEAT1 promotes osteoblast function by upregulating paraspeckles, which promote E3 ubiquitin ligase Smurf1 mRNA retention, thus preventing RUNX2 degradation.^286^ A recent study suggested that exosomes derived from osteoclasts target osteoblasts through ephrinA2/EphA2, and exosomes containing the lncRNA LIOCE facilitate bone formation by upregulating the osteogenic transcription factor Osterix.^287^

Derived from the monocyte/macrophage hematopoietic lineage, osteoclasts are involved in bone resorption. Yang et al.^288^ constructed bioactive glass nanoparticles (BGN) containing EVs derived from BMSCs, which are rich in the lncRNA NRON. NRON suppresses osteoclast differentiation by interacting with the nuclear factor of activated T cells transcription factors and preventing the nuclear translocation of nuclear factor of activated T cell cytoplasmic 1 (NFATc1), a pivotal transcription factor for osteoclastogenesis.^288^ Besides, NFATc1 can be regulated by lncAK077216, which enhances NFATc1 expression and accelerates RANKL-induced osteoclastogenesis and bone resorption by downregulating NIP45.^289^

#### The role of circRNAs in metabolic disease

##### Diabetes mellitus and its complications

circRNAs are implicated in diabetes mellitus and its complications. Recently, Yan et al.^290^ revealed that by sponging miR-93-5p, circ_0071336 increases GLUT4 expression and contributes to the development of T2D. Macrophages are participated in the pathogenesis of T1D, and circPPM1F, a novel circRNA mainly expressed in monocytes, has been reported to act as a positive regulator to activate M1 macrophages, which may exacerbate pancreatic islet injury. circPPM1F expression is elevated in patients with T1D, and circPPM1F overexpression ameliorates the inhibitory effect of protein phosphatase, Mg^2+^/Mn^2+^ dependent 1F (PPM1F) on the NF-κB pathway by binding to human antigen R (HuR). Besides, fused in sarcoma (FUS) and eukaryotic initiation factor 4A-III (EIF4A3) also participate in the process of M1 macrophage activation regulated by circPPM1F.^291^ circRNAs are also responsible for the pathogenesis of GDM. circMAP3K4 and PTPN1 are upregulated in the placentas of patients with GDM, while miR-6795-5p is reduced. Besides, the levels of circMAP3K4 in the placenta of patients with GDM are positively correlated with weight gain during pregnancy. By sequestering miR-6795-5p, circMAP3K4 increases the expression of PTPN1 and inhibits the insulin-PI3K/AKT signalling pathway to regulate insulin resistance in trophoblasts.^292^

Regarding the role of circRNA in complications related to diabetes, Liu et al.^293^ found decreased expression of circNNT and tissue inhibitor of metalloproteinase 3 (TIMP3) and elevated expression of miR-320b in the human retinal pigment epithelial cell line ARPE-19 treated with high glucose. circNNT reportedly prevents the development of DR by protecting ARPE-19 cells against high glucose–induced inflammation and apoptosis through sponging miR-320b and increasing TIMP3.^293^ A study has shown that circHIPK3 is decreased in diabetes and decreased more in diabetic cardiomyopathy (DCM). circHIPK3 overexpression can protect cardiomyocytes from apoptosis evoked by high glucose by downregulating PTEN.^294^ Recently, Meng and colleagues^295^ revealed that circARHGAP12 facilitates diabetic wound healing by promoting the survival of MSCs in diabetic wounds. circARHGAP12 modulates the expression of ATG16L1 and ULK2 by sponging miR-301b-3p, thus accelerating diabetic wound healing.^295^

##### Obesity

circRNAs have been implicated in adipogenesis and adipocyte metabolism. circSAMD4A acts as a regulator of adipogenesis. In obese mice, circSAMD4A interference decreases food intake; restores weight gain; and enhances energy expenditure, glucose tolerance, and insulin sensitivity. By serving as a competitive endogenous RNAs (ceRNA) for miR-138-5p, circSAMD4A elevates enhancer of zeste homolog 2 (EZH2) expression to regulate preadipocyte differentiation.^296^ Besides, circFLT1 serves as an miR-93 sponge and enhances the expression of lncSLC30A9, which binds the FOS protein to the PPARγ promoter to facilitate adipocyte differentiation and suppresses adipocyte proliferation by inactivating the AKT signalling pathway.^297^ Similarly, circPPARγ accelerates adipocyte differentiation and restrains adipocyte proliferation and apoptosis by sponging miR-92a-3p.^298^ On the contrary, a study of cattle adipocytes indicated that circFUT10 facilitates adipocyte proliferation and suppresses adipocyte differentiation by increasing the expression of PGC-1β by interacting with let-7c.^299^ Liu et al.^300^ found that circOgdh is upregulated in BAT, which stimulates the expression of Atgl by acting as a sponge for miR-34a-5p, thus contributing to lipolysis of brown adipocytes and reducing the accumulation of lipid droplets. circTshz2-1 and circArhgap5-2 are increased significantly during adipocyte differentiation. Moreover, knockdown of both these circRNAs suppresses adipocyte differentiation. The results of gene set enrichment analysis suggested that circArhgap5-2 is associated with lipid metabolism and adipocyte differentiation. However, circArhgap5-2 neither encodes new peptides nor sequesters miRNAs, so the mechanism is still unknown.^301^

Adipose inflammation is one of the features of obesity. Zhang et al.^302^ showed that by sponging miR-103, circARF3, also called ADP-ribosylation factor 3, stimulates the expression of TRAF3, which inhibits the NF-κB signalling pathway and promotes mitophagy, thereby restraining the activation of NLRP3 and ameliorating inflammation in adipose tissue.

Taken together, circRNA is not only participated in adipogenesis and adipocyte differentiation, but also responsible for the inflammation in adipose tissue, thereby being a critical regulator in the development of obesity.

##### NAFLD

Lipid metabolism can also be influenced by circRNAs. Li et al.^303^ constructed a nanodrug system to upregulate circRNA_0001805 in hepatocytes, which contributed to treating NAFLD by sponging miR-106a-5p and miR-320a. These miRNAs separately suppress ATP-binding cassette transporter A1 (ABCA1) and carnitine palmitoyl transferase 1 (CPT1), collaboratively inhibiting inflammation and the accumulation of lipids, thus ameliorating NAFLD.^303^ In addition, circ_0057558 is upregulated in NAFLD models, and the study revealed that circ_0057558 sequesters miR-206, which restores the Rho-associated kinase 1 (ROCK1)–AMPK signalling pathway and stimulated lipogenesis and TG secretion, thereby exacerbating NAFLD.^304^ Besides, by sponging miR-188-3p, circ_0048179 increases GPX4 levels and decelerates lipid accumulation.^305^

circRNAs play a pivotal role in liver fibrosis. Zhao et al.^306^ revealed that circRNAs in mitochondria account for the majority of downregulated circRNAs in fibroblasts of patients with NASH. Among these downregulated mitochondrial circRNAs, circRNA ATP5B regulator ameliorates NASH by suppressing fibroblast activation and the output of mitochondrial ROS, which is mediated by PGC-1α and interacted with ATP5B. In addition, lipid overload downregulates PGC-1α by ER stress–induced CHOP.^306^

Regulation of autophagy is also associated with NAFLD. Authors have shown that downregulation of circRNA_002581 significantly decreases the accumulation of lipids and pro-inflammatory cytokines, aspartate aminotransferase (AST) and alanine aminotransferase (ALT) in NASH models, while ATP levels are enhanced. Furthermore, inhibition of circRNA_002581 mitigates NASH by targeting miR-122 and restoring CPEB1, thus promoting autophagy through the CPEB1–PTEN–AMPK–mTOR pathway.^307^

Overall, circRNAs are involved in NAFLD by regulating lipid metabolism and autophagy and by influencing the progression of liver fibrosis. These studies provide a better understanding of mechanisms for NAFLD, but also offer new therapies to treat this condition.

##### Osteoporosis

BMSCs, osteoclasts, and osteoblasts are three vital cells for the process of bone formation and the pathogenesis of osteoporosis. It is widely acknowledged that circRNAs participate in the differentiation of BMSCs. In 2019, Zhang and his colleague^308^ used a microarray to analyse the expression profiles of circRNAs during osteoblast differentiation. They found that 3,938 circRNAs are increased and 1505 are decreased in BMSCs at day 7. Besides, downregulating circIGSF11 can upregulate miR-199b-5p to promote osteoblast differentiation.^308^ Hsa_circ_0074834 can promote osteogenesis–angiogenesis coupling in BMSCs by serving as a sponge for miR-942-5p to increase the expression of VEGF and ZEB1.^309^ BMP2 participates in inducing osteogenic differentiation and BMP2 expression could be upregulated by circRNA_0016624 by sponging miR-98, thus preventing PMOP.^310^ Similarly, circRNA_0048211 prevents PMOP by enhancing BMP2 expression by targeting miR-93-5p.^311^ Recently, circStag1 has been reported to stimulate BMSC osteogenic differentiation. circStag1 interacts with HuR and helps transport this protein to the cytoplasm, which increases the expression of β-catenin and low-density lipoprotein receptor-related protein 5/6 (Lrp5/6), and activates the WNT signalling pathway.^312^

Several studies have explored the role of circRNAs in osteoblasts. circRNA AFF4 serves as a ceRNA for miR-7223-5p and increases the expression of PIK3R1, thus preventing MC3T3-E1-mediated cell apoptosis and promoting osteoblast proliferation.^313^ Liang et al.^314^ increased the expression of circHIPK3 by lentivirus and found that the oxidative injury to human osteoblasts induced by hydrogen peroxide (H_2_O_2_) is decreased. Moreover, by using targeted small hairpin RNA (shRNA), they silenced circHIPK3 and found increased cytotoxicity induced by H_2_O_2_ and miR-124 in primary human osteoblasts.

A few researches concentrated on the function of circRNAs in osteoclasts. circRNA_28313 relieves the suppression on CSF1 by targeting miR-195a, therefore affecting OVX-induced bone absorption in mice. Besides, circRNA_28313 knockdown prevents osteoclast differentiation.^315^ Wang and his colleague recently revealed that a novel circRNA, circBBS9, is involved in the development of osteoporosis.^316^ circBBS9 serves as a natural endogenous sponge of miR-423-3p and thus enhances the expression of TRAF6, which can regulate osteoclasts multinucleation.

Overall, circRNAs are participated in osteoporosis by modulating the differentiation of BMSCs, osteoclasts, and osteoblasts. Given the limited amount of research on the role of circRNAs in osteoporosis, more studies need to be conducted.

### The role of epigenetic regulation in other metabolic diseases

#### Gout

Gout is a common chronic disease that presents with intermittent episodes. Gout is caused by the deposition of MSU crystals; hence, the serum urate concentration is a risk factor to develop the disease.^317^ A study reported that the DNMT1 rs2228611 polymorphism may function in the development of gout, which is increased in patients with gout.^318^ Besides, Wang et al.^319^ explored gout-associated enrichment of differential DNA methylation in adaptive immunity, including pathways for B and T cell receptor signalling, IL-17 signalling, and Th17 development. In another study, researchers found seven DNA methylation sites in patients with gout that map to seven genes, namely PGGT1B, UBAP1, RAPTOR, INSIG1, ANGPTL2, CNTN5, and JNK1.^320^ In a study of gout risk in the male Chinese Han population, authors found that the CCL2 promoter is hypomethylated.^321^ Conversely, the UMOD gene, which encodes the uromodulin glycoprotein, is significantly methylated in patients with gout.^322^ Zhu et al.^323^ indicated that the gout risk gene nuclear receptor binding protein 1 (NRBP1) is overexpressed due to hypomethylation of its promoter region and inhibition of transcription factor AP-2 alpha (TFAP2A) binding. Romidepsin, a dual HDAC1/2 inhibitor, upregulates transcription of suppressor of cytokine signalling 1 (SOCS1), thus contributing to decrease MSU crystal–induced cytokine production.^324^ High UA concentrations can promote IL-1β production in PBMCs; and one of the mechanisms is histone methylation–mediated downregulation of IL-1Ra.^325^

ncRNAs are also involved in the development of gout. Li et al.^326^ revealed that miR-221-5p may relieve acute gouty arthritis (GA) by inhibiting IL-1β expression. Similarly, miR-488 and miR-920 prevent the expression of IL-1β in THP-1 cells by targeting the 3’-UTR of IL-1β and may act as novel potential therapeutic targets for GA.^327^ Liu et al.^328^ knocked down HOTAIR, a lncRNA, and found decreased inflammatory cytokine production via miR-20b upregulation and NLRP3 downregulation. lncRNA‑MM2P, a regulator of M2 polarisation, decreases pro‑inflammatory cytokines and participates in AGA.^329^ circHIPK3 sponges miR-192 and miR-561 and upregulates the expression of TLR4 and NLRP3, thereby contributing to the inflammatory response in GA.^330^ Meng et al.^331^ revealed that total glucosides of paeony (TGP) may be a potential ingredient to treat GA by regulating the MALAT1–miR-876-5p–NLRP3 signalling pathway and the TLR4–MyD88–NF-κB axis.

#### Hyperthyroidism

Hyperthyroidism is characterised by increased thyroid hormone synthesis and secretion which is usually caused by Graves’ disease (GD) and toxic nodular goitre.^332^ Limbach et al.^333^ revealed that dysregulated DNA methylation and histone modifications of T cell signalling genes are involved in the development of GD. Furthermore, newly diagnosed patients with GD have hypomethylation and lower DNMT1 expression in B and T lymphocytes. One study suggested that radioiodine and antithyroid drug treatment would increase DNA methylation and DNMT1 expression to alleviate hyperthyroidism.^334^ In addition, methylation of the IFNG gene is associated with the development of autoimmune thyroid diseases.^335^ Besides, methyltransferase-like 3 (METTL3) is participated in the pathogenesis of GD by inducing m6A modification of SOCS family mRNA.^336^ As a common complication of GD, Graves’ ophthalmopathy (GO) involves hypermethylation genes associated with inflammation and hypomethylated genes related to autoimmunity in orbital fibroblasts.^337,338^ HDAC4 is participated in the pathogenesis of GO by facilitating proliferation and extracellular matrix production in orbital fibroblasts.^339^

There have been several studies regarding the role of ncRNAs in hyperthyroidism and associated disorders. The miRNA let-7b is increased in the serum of patients with untreated GD, which stimulates the expression of thyroid-stimulating hormone receptor (TSHR) in thyroid cells and suppresses the expression of promyelocytic leukaemia zinc finger (PLZF).^340^ The lncRNA LPAL2 activates orbital fibroblasts in patients with thyroid eye disease (TED). LPAL2 interacts with miR-1287-5p and regulates the epidermal growth factor receptor (EGFR)–AKT signalling pathway to increase cell adhesion factor levels.^341^ circRNA_000102 is increased significantly in plasma exosomes of patients with GD. Moreover, circRNA_000102 is involved in the progress of GD via IFNβ signalling and viral infection to activate the immune system.^342^

#### Hypothyroidism

Hypothyroidism is characterised by the deficiency of thyroid hormone; common symptoms are fatigue, weight gain, and cold intolerance.^343^ Luo et al.^344^ performed RNA sequencing and analysed genome-wide DNA methylation. They found that DNA methylation of cell proliferation–associated signalling pathways, such as MAPK, Ras, and WNT, may participate in diabetes-related hypothyroidism. In 2014, Kim et al.^345^ were the first group to show that HDAC inhibitors (HDACi) effectively relieve hypothyroidism. Moreover, the HDAC3 inhibitor could promote histone acetylation and transcription to ameliorate hypothyroidism-induced cerebellar defects.^346^ Hypothyroidism can develop from the evolution of Hashimoto’s thyroiditis (HT), and chronic inflammation mediated by T cells is involved in the process. There is reduced miR-29a-3p in T cells of patients with HT, and the reduction in miR-29a-3p is related to thyroid injury by targeting T-bet, a T helper 1/CD8 T cell transcription factor.^347^ miR-224-5p may be involved in the development of hypothyroidism by targeting deiodinases, which are implicated in the transition of T4 to rT3.^348^ miR-224-5p directly binds to DIO1 and indirectly modulates DIO3 through phosphorylation of the MAPK/ERK pathway.^348^ Besides, miR-125b-5p inhibits the pathogenesis of hypothyroidism by targeting STAT3.^349^ Wang et al.^350^ found that NEAT1 is involved in the impaired endothelial functions in subclinical hypothyroidism (SCH). NEAT1 serves as a sponge for miR-126, disinhibiting the expression of TRAF7, thus exacerbating endothelial apoptosis and impairing vascular function in patients with SCH.^350^

## Interactions between non-genetic risk factors, genetics, and epigenetics in metabolic diseases

**Fig. 5:**
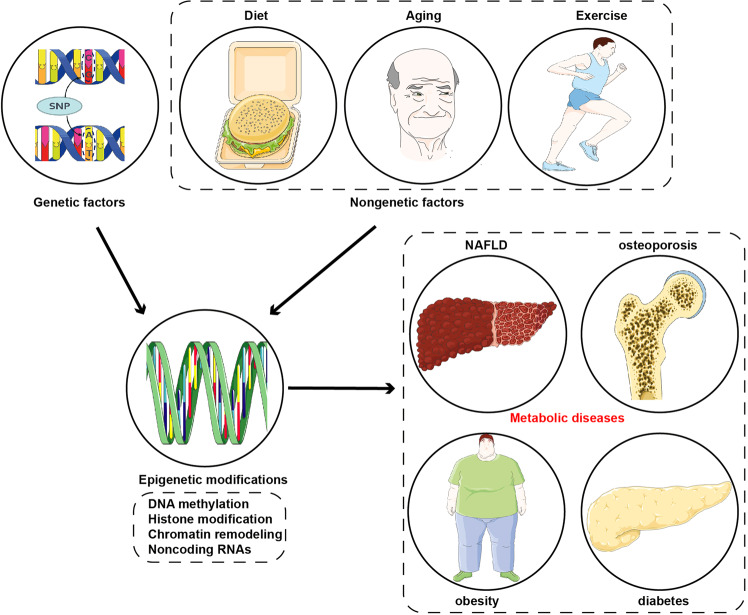
The interactions between nongenetic risk factors, genetics, and epigenetics in metabolic diseases. Both nongenetic risk factors and genetics could affect epigenetics leading to the development of metabolic diseases. This figure was generated with Servier Medical Art (https://smart.servier.com/)

The phenotype is influenced by multiple factors, including nongenetic risk factors, genetics, and epigenetics. These factors work together and interact with each other. Both non-genetic risk factors and genetics could affect epigenetics, leading to the development of metabolic diseases (Fig. 5).

### Interactions between nongenetic risk factors and epigenetics in metabolic diseases

Growing evidence has confirmed that non-genetic risk factors, including ageing, diet, and exercise, can affect epigenetics in metabolic diseases. The concept of the ‘epigenetic clock’ was proposed for the first time in 2013, and the epigenetics of ageing has attracted increased attention since that time.^351^ As a complex process, ageing is related to a decline in physiological functions and usually leads to the elevated prevalence of chronic metabolic diseases, such as osteoporosis and T2D. NDUFB6, a respiratory chain component related to insulin sensitivity, is decreased in skeletal muscle of patients with diabetes. Elderly people present lower NDUFB6 expression and higher DNA methylation levels compared with younger people.^352^ Similarly, COX7A1, another respiratory chain component, is downregulated in skeletal muscle of patients with diabetes. Age affects the expression of COX7A1 and DNA methylation in human skeletal muscle.^353^ In addition, Bacos et al.^354^ indicated that DNA methylation alterations linked to age in human islets are related to T2D. Besides, HOX and RUNX2 present high methylation levels in aged MSCs, which may result in age-related bone loss.^355^

Diet regulates epigenetic alterations and is also a critical factor participated in the pathogenesis of metabolic diseases, such as T2D, NAFLD, and gout. An HFD facilitates the progression of DR by hypermethylating mtDNA and the Rac1 promoter in T2D mice model.^356^ Chen et al.^170^ revealed that maternal consumption of a western-type diet could lead to NAFLD in male offspring by increasing methylation levels of ApoB. In patients with gout, genistein and resveratrol may change DNA methylation levels, whereas curcumin affects miRNA expression.^357^

Exercise and environmental pollution could also result in epigenetic alterations. After first-degree relatives of patients with T2D had exercised, there was decreased expression of several genes, such as MEF2A, NDUFC2, and RUNX1, due to increased methylation levels in skeletal muscle.^358^ In addition, air pollution may lead to metabolic syndrome through DNA methylation changes.^359^

### Interactions between genetics and epigenetics in metabolic diseases

Genetics is a pivotal factor in the development of metabolic diseases. Twin and adoption studies and linkage analyses have provided an abundance of evidence on the role of genetics. Genetics and epigenetics act together on metabolic diseases. In 2013, Dayeh and his colleague^360^ revealed that in patients with T2D, 19 of 40 single nucleotide polymorphisms (SNPs) associated with T2D could affect diabetes-related gene expression through the introduction or removal of a CpG site. In 2014, Olsson et al.^361^ conducted the first genome-wide DNA methylation quantitative trait locus (mQTL) analysis in human pancreatic islets. They identified several methylated SNP-CpG pairs in cis or in trans which are related to insulin secretion. Besides, Shah et al.^362^ revealed that altered methylation levels of SNP rs231840 could change the methylation levels of T2D-related locus KCNQ1, thus influencing insulin sensitivity. By assessing methylation levels changes in adipose tissue from twins, Grundberg et al.^363^ revealed that the BMI SNP rs713586 overlaps an enhancer upstream of adenylate cyclase 3 (ADCY3) and may contribute to obesity. Of interest, compared with unrelated people or same-sex dizygotic twins, genome-wide DNA methylation levels of adipose tissue present a stronger correlation in monozygotic twins.^364^ In addition, there are different DNA methylation levels in skeletal muscle between individuals with and without a family history of T2D.^358^ Taken together, gene expression is modulated by epigenetic mechanisms and contributes to the development of metabolic diseases.

## Clinical applications of epigenetics in metabolic diseases

### Epigenetic biomarkers

Epigenetic biomarkers play a vital role in the early diagnosis and prognosis of metabolic diseases (Table 5). DNA methylation biomarkers have been well studied. The methylation level of PHOSPHO1 is negatively correlated with the risk of T2D, while the methylation level of ABCG1 is associated with an elevated risk of T2D.^365^ In addition, methylation in TXNIP, SREBF1, and SOCS3 is related to the incidence of T2D.^366^ Johnson et al.^367^ identified seven CpG sites, including cg09822959 and cg19686543, which are related to liver fibrosis and could act as promising biomarkers for liver fibrosis. Besides, methylation levels of the mitochondrially encoded NADH dehydrogenase 6 (MT-ND6) are closely associated with the severity of NAFLD.^368^ For patients with obesity, the beta-3 adrenoceptor (ADRB3) gene presents high methylation levels in WAT and it contributes to the susceptibility to obesity.^369^ In patients with PMOP, researchers identified 77 significantly differentially methylated CpG sites, including ZNF267 and ABLIM2, and they may act as biomarkers for the diagnosis of osteoporosis.^177^

**Table 5:** Epigenetic biomarkers in the clinical setting

Epigenetic biomarkers	Related epigenetic regulation	Disease	Reference
PHOSPHO1	DNA methylation	T2D	^365^
ABCG1	DNA methylation	T2D	^366^
TXNIP, SREBF1, and SOCS3	DNA methylation	T2D	^366^
cg09822959, cg19686543	DNA methylation	NAFLD	^367^
MT-ND6	DNA methylation	NAFLD	^368^
ADRB3	DNA methylation	obesity	^369^
ZNF267, ABLIM2	DNA methylation	osteoporosis	^177^
miR-21-5p, miR-29a-3p, let-7b-5p, and let-7c-5p	miRNA	ESRD in patients with T1D	^370^
miR-155	miRNA	T1D	^371^
miR-21	miRNA	IGT and prediabetic status	^372^
miR-3659	miRNA	Dyslipidaemia and obesity	^373^
miR‑135a‑3p	miRNA	NAFLD	^375^
miR-33a	miRNA	NAFLD	^374^
miR-148a and miR-122-5p	miRNA	osteoporosis	^376^
hsa_circ_0076690	circRNA	osteoporosis	^377^

ncRNAs, especially miRNAs, could also act as epigenetic biomarkers in metabolic diseases. This potentiality is attributed to the availability of acquisition and quantitative analyses, as well as stability in biofluids and exosomes. For example, Pezzolesi et al.^370^ indicated that circulating miRNAs regulated by TGF-β1, including miR-21-5p, miR-29a-3p, let-7b-5p, and let-7c-5p, could act as biomarkers for rapid progression of end-stage renal disease (ESRD) in patients with T1D. After controlling other covariates, they found that miR-21-5p and let-7b-5p contributed to a more than 2.5-fold increase in the risk of ESRD (*p* ≤ 0.005), whereas miR-29a-3p and let-7c-5p were related to a marked decrease in the risk of rapid progression (*p* ≤ 0.001).^370^ The receiver operating characteristic (ROC) curve of patients with T1D indicated that miR-155 could serve as a biomarker for patients with T1D; the area under the ROC (AUC) of miR-155 was 0.73.^371^ In addition, miR-21 presented a predictive value for impaired glucose tolerance (IGT) and prediabetic status, as the AUC of miR-21 was 0.8 (p = 0.0004) in discriminating IGT from normal glucose tolerance (NGT).^372^ In patients with obesity, circulating miR-3659 may act as a possible biomarker of dyslipidaemia with an AUC of 0.806.^373^ Besides, circulating miR‑135a‑3p in serum EVs and circulating miR-33a are potential biomarkers for NAFLD.^374,375^ Other biomarkers of osteoporosis may include hsa_circ_0076690, miR-148a, and miR-122-5p.^376,377^

### Epigenetic therapy

**Fig. 6:**
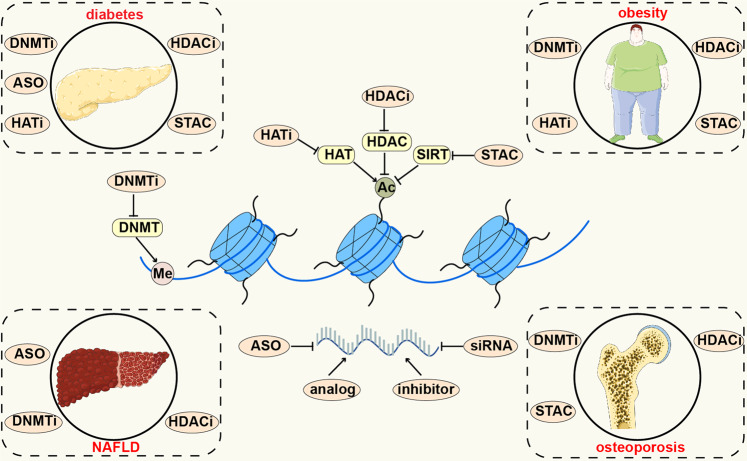
Therapeutic application of epigenetics. The figure presented epigenetics-related targets and drugs. This figure was generated with Servier Medical Art (https://smart.servier.com/)

As the study of epigenetics has advanced, a series of epigenetic therapies targeted to different epigenetic mechanisms have been developed (Fig. 6). Currently, no epigenetic drug has been approved by the U.S. FDA for the treatment of metabolic diseases. Meanwhile, several epigenetic drugs approved for other diseases have been reported to be involved in metabolic diseases. In Table 6 and Table 7, we separately summarise potential epigenetic drugs for metabolic diseases and several clinical trials extracted from https://clinicaltrials.gov, respectively.

**Table 6:** Potential epigenetic drugs for the treatment of metabolic disease

Drugs	Target gene	Diseases	References
**DNMTi**			
hydralazine	Nrf2	diabetic nephropathy	^378^
procainamide	PDX1	diabetes	^381^
decitabine	PPARγ	diabetes	^382^
5-aza-2’-deoxycytidine	PPARγ1	obesity	^383^
5-aza-2’-deoxycytidine	PPAR-α	NAFLD	^384^
5-aza-2’-deoxycytidine	OPN, RUNX2	diabetic osteoporosis	^385^
**HDACi**			
VPA	STAT5	T1D	^387^
VPA	RUNX2	osteoporosis	^388^
vorinostat	OPG	diabetes	^389^
vorinostat	EGFR	diabetic nephropathy	^390,391^
SAHA	Zfp719	obesity	^392^
SAHA	insulin receptor β, Akt, and FoxO1	osteoporosis	^393^
Givinostat	Gata3, FOXP3, IL-6, IL-12, TNF-α	diabetes	^397^
Givinostat	IL-1β, IL-6 and TNF-α	diabetes	^398^
Givinostat	IL-6, IL-1β, TNF-α	nonalcoholic steatohepatitis	^399^
Dacinostat	UCP1, Ppargc1α	obesity	^400^
TSA	AMPK	obesity	^401^
Puerarin	HDAC1/HDAC3	diabetic osteoporosis	^403^
**HATi**			
Curcumin	FOXO1	DCM	^405^
Curcumin	HSP-27, p38	DN	^406^
C66	CTGF, PAI-1 and FN-1	DN	^407^
C646	IRS1/2	diabetes, obesity	^408^
**STAC**			
resveratrol	SIRT1, NF-kB-p65	cardiac oxidative stress in diabetes	^409^
resveratrol	SIRT1, PGC-1α	Obesity, diabetic cardiomyopathy	^410,411^
resveratrol	SIRT1, FOXO3a	DN	^412^
resveratrol	SIRT1, FOXO 1	osteoporosis	^413^
HPE	SIRT1, NF-kB, p53	DN	^414^
**ASO**			
IONIS-GCGRRx	GCGR	T2D	^416^
Vupanorsen	ANGPTL3	hepatic steatosis, diabetes	^417^

**Table 7:** Clinical trials with epigenetics drugs in metabolic disease

Drug	Disease	Study type	Number of participants	Recruitment status/phase	NCT number
**DNMTi**					
Hydralazine	T2D	Interventional	10251	Phase 3	NCT00000620
**HDACi**					
Valproic acid	Obesity	Interventional	22	Phase 4	NCT00298857
sodium phenylbutyrate	Diabetes	Interventional	10	Phase 4	NCT00533559
sodium phenylbutyrate	Diabetes, obesity	Interventional	101	Not Applicable	NCT00771901
sodium phenylbutyrate	Obese non diabetic	Interventional	6	Not Applicable	NCT05028803
ricolinostat	Painful Diabetic Peripheral Neuropathy	Interventional	282	Phase 2	NCT03176472
**HATi**					
Curcumin	T2D	Interventional	200	Phase 4	NCT01052597
Curcumin	T2D	Interventional	60	Phase 4	NCT04528212
Curcumin	T2D	Interventional	50	Phase 2/3	NCT02529969
Curcumin	T2D	Interventional	44	Not Applicable	NCT02529982
Curcumin	T2D, obeisty	Interventional	15	Not Applicable	NCT03542240
Curcumin	T2D, NAFLD	Interventional	50	Phase 2/3	NCT02908152
Curcumin	Prediabetes	Interventional	142	Phase 4	NCT03917784
Curcumin	Obesity, NAFLD	Interventional	39	Not Applicable	NCT03864783
Curcumin	NAFLD	Interventional	24	Not Applicable	NCT04315350
**STAC**					
Resveratrol	Prediabetes	Interventional	42	Not Applicable	NCT02565979
Resveratrol	T2D	Interventional	20	Phase 2	NCT01354977
Resveratrol	T2D	Interventional	22	Phase 2	NCT02549924
Resveratrol	Prediabetes	Interventional	15	Not Applicable	NCT02129595
Resveratrol	Gestational diabetes	Interventional	112	Phase 4	NCT01997762
Resveratrol	Diabetic nephropathy	Interventional	60	Early phase 1	NCT02704494
Resveratrol	Obesity, NAFLD	Interventional	26	Not Applicable	NCT01446276
Resveratrol	Obesity, osteoporosis	Interventional	76	Not Applicable	NCT01412645
Resveratrol	NAFLD	Interventional	50	Phase 2/3	NCT02030977
**antisense oligonucleotide**					
ISIS-GCGRRx	T2D	Interventional	77	Phase 2	NCT01885260
ISIS-GCGRRx	T2D	Interventional	79	Phase 2	NCT02583919
ISIS-GCGRRx	Diabetes	Interventional	10	Phase 4	NCT02824003
ISIS 703802	T2D, NAFLD	Interventional	105	Phase 2	NCT03371355

#### DNA methylation

DNA methyltransferase inhibitors (DNMTi) modulate the methylation levels of specific genes, providing a strategy for the treatment of metabolic diseases. The U.S. FDA has approved some epigenetic drugs acting as DNMTi, including azacytidine and guadecitabine. However, none of them have been approved for application in metabolic diseases. Notably, several DNMTi approved for other diseases may also function in metabolic diseases, including hydralazine, procainamide, and decitabine.

Acting as a smooth muscle relaxant, hydralazine is applied for the treatment of hypertension. Hydralazine could play a protective role in DKD in patients with diabetes by preventing ROS production via xanthine oxidase (XO) inhibition and activating Nrf2-mediated haem oxygenase 1 (HO-1).^378^ Besides, low-dose hydralazine alleviates obesity-related chronic kidney disease; the potential mechanism is by reducing the obesity-induced methylation levels in the whole kidney.^379^ Procainamide is an antiarrhythmic drug and partial competitive inhibitor of DNMT1.^380^ Procainamide protects against diabetes in unconventional prefoldin RPB5 interactor (URI) deficiency mice by restoring PDX1 expression via a decrease in its methylation levels.^381^ Decitabine, also called 5-aza-2′-deoxycytidine, is the strongest known DNMTi. It specifically works during the S-phase of the cell cycle and is approved for the treatment of myelodysplastic syndromes (MDS). Decitabine could accelerate M1 to M2 macrophage polarisation by targeting PPARγ, which enhances the anti-diabetic effect of UC-MSCs.^382^ Decitabine also suppresses PPARγ1 promoter DNA methylation to facilitate macrophage activation and to restrain insulin resistance in obesity.^383^ Moreover, decitabine increases the expression of PPARα mRNA by regulating DNA methylation levels and reducing lipid accumulation, thereby alleviating NAFLD.^384^ Another study revealed that DNA methylation may act as a promising target for treating diabetic osteoporosis. Decitabine could decrease the methylation levels of osteogenic genes, including OPN and RUNX2, and suppress WNT–β-catenin signalling pathway to facilitate the osteogenic differentiation of adipose-derived stromal cells (ASCs).^385^

#### Histone modifications

Histone modification is an attractive strategy for possible clinical interventions and several related drugs have been developed, including HDACi, HAT inhibitors (HATi), and sirtuin-activating compounds (STAC).

##### HDACi

There has been rapid development of HDACi over the years. Several HDACi have been approved by U.S. FDA, including valproic acid (VPA), vorinostat, and sodium phenylbutyrate. VPA is an antiepileptic drug that is reported to be involved in diabetes. VPA promotes diabetes-associated reduced acetylation of H3 in the pancreas and suppress β cell apoptosis.^386^ Besides, VPA increases the expression of STAT5 and H3 acetylation to promote Treg differentiation, thereby inhibiting autoimmune reactions in islet transplantation and contributing to the treatment for T1D.^387^ VPA could promote the differentiation of osteoblasts and bone formation by elevating the expression of RUNX2 through hyperacetylation of histone H3.^388^ Vorinostat, also known as suberoylanilide hydroxamic acid (SAHA), is approved for the treatment of cutaneous T cell lymphoma. Vorinostat increases histone acetylation of the OPG locus in osteoblasts and promotes OPG transcription, thus increasing systemic insulin sensitivity.^389^ Studies have revealed that vorinostat alleviates renal damage in a mouse model of diabetes by decreasing EGFR or endothelial nitric oxide synthase (eNOS).^390,391^ Vorinostat enhances histone H3 acetylation at the zinc finger protein 719 (Zfp719) promoter and increases the expression of Zfp719 and UCP1, which promotes white fat browning and lipid catabolism in adipocytes.^392^ By increasing the acetylation of histone H4, vorinostat upregulates insulin signalling regulators and reduces the phosphorylation of insulin receptor β, AKT, and FOXO1. These changes facilitate the differentiation of terminal osteoblasts.^393^ Sodium phenylbutyrate or 4-phenyl butyric acid (PBA) acts as an HDACi and chemical chaperone associated with ER stress; it is approved for the treatment for urea cycle disorders. PBA attenuates β cell dysfunction and insulin resistance by decreasing ER stress, providing a potential treatment strategy for diabetes.^394,395^

There are other HDACi could also serve as potential drugs to treat metabolic diseases, although they have not yet received U.S. FDA approval. Givinostat is a lysine deacetylase inhibitor (KDACi). Givinostat could prevent diabetes by protecting β cells against from inflammatory damage.^396^ Givinostat increases Treg subsets, upregulates the transcription factors Gata3 and FOXP3, and reduces inflammatory dendritic cell subsets and the cytokines IL-6, IL-12, and TNF-α, thereby ameliorating diabetes.^397^ Furthermore, the combination of givinostat and low-dose CD3 antibodies can effectively alleviate diabetes by downregulating IL-1β, IL-6, and TNF-α.^398^ Similarly, givinostat ameliorates NASH by suppressing the inflammatory cytokines IL-6, IL-1β, and TNF-α.^399^ By activating UCP1 and PGC-1α transcription in adipose tissue via acetylation of histone 3 lysine 27, dacinostat promotes adipose thermogenesis and may act as a potential strategy for treating obesity.^400^ Trichostatin A (TSA) is a novel HDACi and reportedly inhibits adipogenesis in 3T3-L1 preadipocytes through the AMPK signalling pathway.^401^ By inhibiting HDAC and increasing insulin sensitivity, sodium acetate alleviates hepatic lipotoxicity related to diabetes.^402^ Puerarin may attenuate diabetic osteoporosis by preventing inflammation and apoptosis through HDAC1 and HDAC3 inhibition.^403^

##### HATi and STAC

A few HATi have been studied in metabolic diseases, but none of them have been approved by the U.S. FDA. Curcumin is isolated from turmeric, a traditional Chinese medicine.^404^ Curcumin suppresses FOXO1 acetylation and ameliorates DCM by reducing oxidative stress and inhibiting cardiomyocyte apoptosis.^405^ Besides, curcumin decelerates the development of DKD by increasing acetylation of histone H3 and decreasing the expression of HSP-27 and p38.^406^ Moreover, the curcumin analogue C66 decreases the acetylation levels of H3K9/14 at the CTGF, PAI-1, and FN-1 gene promoters to prevent DKD.^407^ C646 is a specific inhibitor of P300 acetyltransferase; it suppresses the acetylation of IRS1/2 and promotes IRS1/2 membrane translocation, resulting in insulin signalling pathway activation.^408^

Resveratrol is a natural polyphenolic compound and could be used to treat metabolic diseases by activating SIRT1. Bagul et al.^409^ indicated that resveratrol alleviates cardiac oxidative stress in diabetes by activating SIRT1, which deacetylates H3 at lysine 9 and NF-κB-p65 at lysine 310. Besides, resveratrol promotes the deacetylation of PGC-1α mediated by SIRT1 to improve mitochondrial function and mitigate metabolic diseases, such as obesity and DCM.^410,411^ Resveratrol could alleviate renal tubular damage induced by hyperglycaemia by deacetylating FOXO3a through SIRT1.^412^ By activating the SIRT1–FOXO1 signalling pathway, resveratrol attenuates bone loss and facilitates osteogenesis in osteoporosis mice.^413^ Hawthorn polyphenol extract (HPE) also activates SIRT1 and ameliorates hyperglycaemia-induced retinal damage by suppressing inflammation and apoptosis in ARPE-19 cells via AMPK–SIRT1–NF-κB pathway activation and miR-34a–SIRT1–p53 pathway suppression.^414^ Of note, in 2007 Milne et al.^415^ identified small molecule activators of SIRT1, including SRT1720, which improve insulin sensitivity and decrease plasma glucose, providing a potential treatment for T2D. These small molecule activators of SIRT1 are not structurally similar to resveratrol and are 1,000-fold more potent.^415^

#### ncRNA

Given their pivotal role in epigenetics, ncRNAs are also interesting targets for treating metabolic diseases. Several treatment strategies have been developed, such as small interfering RNA (siRNA), antisense oligonucleotides (ASO), and analogues or inhibitors of ncRNAs. However, there are few related drugs that have been developed and applied.

IONIS-GCGRRx is a 2′-O-methoxyethyl ASO. Clinical trials (NCT01885260, NCT02583919, and NCT02824003) have confirmed that IONIS-GCGRRx is beneficial for glycaemic control when combined with low weekly doses of metformin. It targets the glucagon receptor (GCGR) in the liver, thus contributing to ameliorating T2D.^416^ Vupanorsen is an *N*-acetyl galactosamine-conjugated ASO that selectively reduces production of angiopoietin-like 3 (ANGPTL3) in the liver by targeting its mRNA. Further studies reported that vupanorsen decreases atherogenic lipoproteins and TG in patients with hepatic steatosis and diabetes.^417^ Berberine (BBR), a constituent of traditional Chinese medicine, presents a promising therapeutic potential for NAFLD. Yuan et al.^418^ revealed that BBR alters the expression of 881 mRNAs and 538 lncRNAs in the steatotic liver, including lncRNAMLAK052686, which is associated with Nrf2.

### Epigenetic editing

Epigenetic editing is used to reprogramme transcription by rewriting the local epigenetic landscape of an endogenous genomic site.^419^ With the progress made in CRISPR/Cas9 and other epigenetic editing tools, epigenetic editing has advanced markedly.^420^ Researchers have reported on epigenetic editing in metabolic diseases. Imprinting control region 2 (ICR2) regulates the cell cycle inhibitor p57 encoded by the CDKN1C gene. Ou et al.^421^ used transcription activator-like effector (TALE) epigenome editing to downregulate the methylation levels of the ICR2 in β cells of human islets. This approach suppresses p57 expression and promotes β cell proliferation and may represent a treatment for diabetes. In addition, Lia and colleagues^422^ increased the levels of pancreatic and duodenal homeobox gene 1 (Pdx1) in liver cells through the CRISPR/Cas9 TGA system, thereby inducing the generation of insulin-producing cells transformed from liver cells. Transient neonatal diabetes mellitus type 1 (TNDM) is a rare disease caused by the overexpression of pleomorphic adenoma gene-like 1 (PLAGL1) and untranslated HYMAI mRNA. By upregulating methylation levels of the HYMA1 promoter through dCas9-DNMT3A, HYMAI expression could be reduced to normal levels and alleviate TNDM.^423^ Claussnitzer et al.^424^ edited rs1421085 using CRISPR-Cas9 to repair the ARID5B motif in primary adipocytes. This editing activated adipocyte browning and promoted thermogenesis. Taken together, there is great therapeutic potential for epigenetic editing as it modulates the expression of genes associated with disease by reprogramming stable epigenetic marks.

## Perspectives and conclusions

In the review, we highlighted the role of epigenetics in metabolic diseases. First, we reviewed the history of epigenetics and briefly introduce some methods and common techniques for the study of epigenetics. Then, we introduced the mechanisms of DNA methylation, histone modification, chromatin remodelling, and ncRNA and their roles in metabolic diseases. It is well known that phenotype is influenced by heredity, epigenetics, and the environment, so we also discussed the interaction between epigenetics and genetics or non-genetic factors in metabolic diseases. Unlike genetics, epigenetic alterations are mostly reversible; hence, epigenetics has great potential clinical application. Several epigenetic biomarkers have been discovered; at the same time, some epigenetic drugs have been developed and epigenetic editing could contribute substantially to treat metabolic diseases. In addition, we summarised the associated clinical trials.

With the application of bioinformatics and rich publicly available datasets, as well as the progress in the available technologies, such as gene editing and high-throughput sequencing, the study of epigenetics has progressed rapidly. Although there have been many studies on epigenetics in metabolic diseases in recent years, there are still many challenges worth exploring. Although chromatin remodelling is a major form of epigenetic regulations, few studies have focused on its role in metabolic diseases. A series of drugs have been developed for DNA methylation and histone acetylation. Though there are multiple studies for ncRNAs and other histone modifications, few of them have been applied in clinical medicine. Till now, none of epigenetic drugs have been approved for metabolic diseases. Furthermore, these epigenetic drugs may have negative effects by altering off-target genes.^425^ So, it is important for us to further study the role of epigenetic drugs in metabolic diseases. Besides, histone modifications are different in various tissues, more researches are needed to explore it. Moreover, the interaction between epigenetics and other non-genetic risk factors or genetics are worth studying.

An in-depth understanding of the epigenetic mechanisms in metabolic diseases is crucial to offer novel ideas and programmes for the prevention, diagnosis, and treatment of metabolic diseases. There are great differences between individuals and small alterations could accumulate to make a big difference due to the heterogeneity of metabolic diseases. There is a great need to develop more epigenetic drugs and related technologies for metabolic diseases, endeavours that require broader cooperation. This is an area with great potential, and many problems still need to be explored.

## References

[1] Fan Y, Pedersen O (2021). Gut microbiota in human metabolic health and disease. Nat. Rev. Microbiol.

[2] Stower H (2019). Personalizing metabolic disease therapies. Nat. Med.

[3] Jaacks LM (2019). The obesity transition: stages of the global epidemic. Lancet Diabetes Endocrinol..

[4] Zheng Y, Ley SH, Hu FB (2018). Global aetiology and epidemiology of type 2 diabetes mellitus and its complications. Nat. Rev. Endocrinol..

[5] Younossi ZM (2016). Global epidemiology of nonalcoholic fatty liver disease-Meta-analytic assessment of prevalence, incidence, and outcomes. Hepatology.

[6] Black DM, Rosen CJ (2016). Clinical Practice. Postmenopausal Osteoporosis. N. Engl. J. Med.

[7] Dalbeth N, Merriman TR, Stamp LK (2016). Gout. Lancet.

[8] Ross DS (2016). 2016 American Thyroid Association Guidelines for Diagnosis and Management of Hyperthyroidism and Other Causes of Thyrotoxicosis. Thyroid.

[9] Persani L (2012). Clinical review: Central hypothyroidism: pathogenic, diagnostic, and therapeutic challenges. J. Clin. Endocrinol. Metab..

[10] Boulton A (2020). Strengthening the International Diabetes Federation (IDF). Diabetes Res Clin. Pr..

[11] Koenen M, Hill MA, Cohen P, Sowers JR (2021). Obesity, Adipose Tissue and Vascular Dysfunction. Circ. Res.

[12] Pan XF, Wang L, Pan A (2021). Epidemiology and determinants of obesity in China. Lancet Diabetes Endocrinol..

[13] Powell EE, Wong VW, Rinella M (2021). Non-alcoholic fatty liver disease. Lancet.

[14] Li J (2019). Prevalence, incidence, and outcome of non-alcoholic fatty liver disease in Asia, 1999-2019: a systematic review and meta-analysis. Lancet Gastroenterol. Hepatol..

[15] Singh JA, Gaffo A (2020). Gout epidemiology and comorbidities. Semin Arthritis Rheum..

[16] Hoffman DJ, Powell TL, Barrett ES, Hardy DB (2021). Developmental origins of metabolic diseases. Physiol. Rev..

[17] Ling C, Ronn T (2019). Epigenetics in Human Obesity and Type 2 Diabetes. Cell Metab..

[18] Chiou J (2021). Interpreting type 1 diabetes risk with genetics and single-cell epigenomics. Nature.

[19] Muraca, M. & Cappariello, A. The Role of Extracellular Vesicles (EVs) in the Epigenetic Regulation of Bone Metabolism and Osteoporosis. *Int J Mol Sci*. **21**, 4923 (2020). 10.3390/ijms21228682 PMC7698531 33213099

[20] Rohde K (2019). Genetics and epigenetics in obesity. Metabolism.

[21] Eslam M, Valenti L, Romeo S (2018). Genetics and epigenetics of NAFLD and NASH: Clinical impact. J. Hepatol..

[22] Shi Y (2022). Epigenetic regulation in cardiovascular disease: mechanisms and advances in clinical trials. Signal Transduct. Target Ther..

[23] Hogg SJ, Beavis PA, Dawson MA, Johnstone RW (2020). Targeting the epigenetic regulation of antitumour immunity. Nat. Rev. Drug Disco..

[24] Nacev BA (2020). The epigenomics of sarcoma. Nat. Rev. Cancer.

[25] Park J, Lee K, Kim K, Yi SJ (2022). The role of histone modifications: from neurodevelopment to neurodiseases. Signal Transduct. Target Ther..

[26] Li X (2022). Lipid metabolism dysfunction induced by age-dependent DNA methylation accelerates aging. Signal Transduct. Target Ther..

[27] Guo L, Lee YT, Zhou Y, Huang Y (2022). Targeting epigenetic regulatory machinery to overcome cancer therapy resistance. Semin Cancer Biol..

[28] Bagert JD, Muir TW (2021). Molecular Epigenetics: Chemical Biology Tools Come of Age. Annu Rev. Biochem.

[29] Phillips RE, Soshnev AA, Allis CD (2020). Epigenomic Reprogramming as a Driver of Malignant Glioma. Cancer Cell.

[30] He X, Kuang G, Wu Y, Ou C (2021). Emerging roles of exosomal miRNAs in diabetes mellitus. Clin. Transl. Med.

[31] Mengozzi, A. et al. Targeting SIRT1 Rescues Age- and Obesity-Induced Microvascular Dysfunction in Ex Vivo Human Vessels. *Circ. Res.* **131**, 476–491 (2022). 10.1161/CIRCRESAHA.122.320888 PMC9426744 35968712

[32] Ling C, Bacos K, Ronn T (2022). Epigenetics of type 2 diabetes mellitus and weight change - a tool for precision medicine?. Nat. Rev. Endocrinol..

[33] Lee HS, Park T (2020). The influences of DNA methylation and epigenetic clocks, on metabolic disease, in middle-aged Koreans. Clin. Epigenet..

[34] Kato M, Natarajan R (2019). Epigenetics and epigenomics in diabetic kidney disease and metabolic memory. Nat. Rev. Nephrol..

[35] Waddington CH (1942). The epigenotype. Int J. Epidemiol..

[36] Waddington, C. H. *The Strategy of the Genes*: *A Discussion of Some Aspects of Theoretical Biology* (George Allen and Unwin, London, 1957)*.*

[37] Hotchkiss RD (1948). The quantitative separation of purines, pyrimidines, and nucleosides by paper chromatography. J. Biol. Chem..

[38] Holliday R, Pugh JE (1975). DNA modification mechanisms and gene activity during development. Science.

[39] Razin A, Riggs AD (1980). DNA methylation and gene function. Science.

[40] Allfrey VG, Faulkner R, Mirsky AE (1964). ACETYLATION AND METHYLATION OF HISTONES AND THEIR POSSIBLE ROLE IN THE REGULATION OF RNA SYNTHESIS. Proc. Natl Acad. Sci. USA.

[41] Kornberg RD (1974). Chromatin structure: a repeating unit of histones and DNA. Science.

[42] Sanger HL (1976). Viroids are single-stranded covalently closed circular RNA molecules existing as highly base-paired rod-like structures. Proc. Natl Acad. Sci. USA.

[43] Brannan CI, Dees EC, Ingram RS, Tilghman SM (1990). The product of the H19 gene may function as an RNA. Mol. Cell Biol..

[44] Lee RC, Feinbaum RL, Ambros V (1993). The C. elegans heterochronic gene lin-4 encodes small RNAs with antisense complementarity to lin-14. Cell.

[45] Luger K (1997). Crystal structure of the nucleosome core particle at 2.8 A resolution. Nature.

[46] Brownell JE (1996). Tetrahymena histone acetyltransferase A: a homolog to yeast Gcn5p linking histone acetylation to gene activation. Cell.

[47] Taunton J, Hassig CA, Schreiber SL (1996). A mammalian histone deacetylase related to the yeast transcriptional regulator Rpd3p. Science.

[48] Rea S (2000). Regulation of chromatin structure by site-specific histone H3 methyltransferases. Nature.

[49] Wu G (2012). Somatic histone H3 alterations in pediatric diffuse intrinsic pontine gliomas and non-brainstem glioblastomas. Nat. Genet.

[50] Schwartzentruber J (2012). Driver mutations in histone H3.3 and chromatin remodelling genes in paediatric glioblastoma. Nature.

[51] Kundaje A (2015). Integrative analysis of 111 reference human epigenomes. Nature.

[52] Nakato R, Shirahige K (2017). Recent advances in ChIP-seq analysis: from quality management to whole-genome annotation. Brief. Bioinform.

[53] Furey TS (2012). ChIP-seq and beyond: new and improved methodologies to detect and characterize protein-DNA interactions. Nat. Rev. Genet.

[54] Gomez D, Shankman LS, Nguyen AT, Owens GK (2013). Detection of histone modifications at specific gene loci in single cells in histological sections. Nat. Methods.

[55] Liu Y (2019). A practical guide for DNase-seq data analysis: from data management to common applications. Brief. Bioinform.

[56] Yan F, Powell DR, Curtis DJ, Wong NC (2020). From reads to insight: a hitchhiker’s guide to ATAC-seq data analysis. Genome Biol..

[57] Shen Y, Chen LL, Gao J (2021). CharPlant: A De Novo Open Chromatin Region Prediction Tool for Plant Genomes. Genom. Proteom. Bioinforma..

[58] Chereji RV, Bryson TD, Henikoff S (2019). Quantitative MNase-seq accurately maps nucleosome occupancy levels. Genome Biol..

[59] Farrell, C. et al. BiSulfite Bolt: A bisulfite sequencing analysis platform. *Gigascience*. **10**, giab033 (2021). 10.1093/gigascience/giab033 PMC8106542 33966074

[60] Booth MJ (2012). Quantitative sequencing of 5-methylcytosine and 5-hydroxymethylcytosine at single-base resolution. Science.

[61] Xu C, Corces VG (2019). Resolution of the DNA methylation state of single CpG dyads using in silico strand annealing and WGBS data. Nat. Protoc..

[62] Lu X (2013). Chemical modification-assisted bisulfite sequencing (CAB-Seq) for 5-carboxylcytosine detection in DNA. J. Am. Chem. Soc..

[63] Kaya-Okur HS (2019). CUT&Tag for efficient epigenomic profiling of small samples and single cells. Nat. Commun..

[64] Bartosovic M, Kabbe M, Castelo-Branco G (2021). Single-cell CUT&Tag profiles histone modifications and transcription factors in complex tissues. Nat. Biotechnol..

[65] Skene, P. J. & Henikoff, S. An efficient targeted nuclease strategy for high-resolution mapping of DNA binding sites. *Elife*. **6**, e21856 (2017). 10.7554/eLife.21856 PMC5310842 28079019

[66] Ma X, Kang S (2019). Functional Implications of DNA Methylation in Adipose Biology. Diabetes.

[67] Nishiyama A, Nakanishi M (2021). Navigating the DNA methylation landscape of cancer. Trends Genet.

[68] Dawson MA, Kouzarides T (2012). Cancer epigenetics: from mechanism to therapy. Cell.

[69] Kriaucionis S, Heintz N (2009). The nuclear DNA base 5-hydroxymethylcytosine is present in Purkinje neurons and the brain. Science.

[70] Tahiliani M (2009). Conversion of 5-methylcytosine to 5-hydroxymethylcytosine in mammalian DNA by MLL partner TET1. Science.

[71] Maiti A, Drohat AC (2011). Thymine DNA glycosylase can rapidly excise 5-formylcytosine and 5-carboxylcytosine: potential implications for active demethylation of CpG sites. J. Biol. Chem..

[72] Lyko F (2018). The DNA methyltransferase family: a versatile toolkit for epigenetic regulation. Nat. Rev. Genet.

[73] Chen Z, Zhang Y (2020). Role of Mammalian DNA Methyltransferases in Development. Annu Rev. Biochem.

[74] Langan TA (1968). Histone phosphorylation: stimulation by adenosine 3’,5’-monophosphate. Science.

[75] Lepack AE (2020). Dopaminylation of histone H3 in ventral tegmental area regulates cocaine seeking. Science.

[76] West MH, Bonner WM (1980). Histone 2B can be modified by the attachment of ubiquitin. Nucleic Acids Res.

[77] Zhao Y, Garcia BA (2015). Comprehensive Catalog of Currently Documented Histone Modifications. Cold Spring Harb. Perspect. Biol..

[78] Shvedunova M, Akhtar A (2022). Modulation of cellular processes by histone and non-histone protein acetylation. Nat. Rev. Mol. Cell Biol..

[79] Marmorstein R, Zhou MM (2014). Writers and readers of histone acetylation: structure, mechanism, and inhibition. Cold Spring Harb. Perspect. Biol..

[80] Ogryzko VV (1996). The transcriptional coactivators p300 and CBP are histone acetyltransferases. Cell.

[81] Li G, Tian Y, Zhu WG (2020). The Roles of Histone Deacetylases and Their Inhibitors in Cancer Therapy. Front Cell Dev. Biol..

[82] Seto E, Yoshida M (2014). Erasers of histone acetylation: the histone deacetylase enzymes. Cold Spring Harb. Perspect. Biol..

[83] Dhalluin C (1999). Structure and ligand of a histone acetyltransferase bromodomain. Nature.

[84] Filippakopoulos P (2012). Histone recognition and large-scale structural analysis of the human bromodomain family. Cell.

[85] Cochran AG, Conery AR, Sims RJ (2019). Bromodomains: a new target class for drug development. Nat. Rev. Drug Disco..

[86] Wang N, Wu R, Tang D, Kang R (2021). The BET family in immunity and disease. Signal Transduct. Target Ther..

[87] Gong F, Miller KM (2019). Histone methylation and the DNA damage response. Mutat. Res Rev. Mutat. Res.

[88] Santos-Rosa H (2002). Active genes are tri-methylated at K4 of histone H3. Nature.

[89] Wagner EJ, Carpenter PB (2012). Understanding the language of Lys36 methylation at histone H3. Nat. Rev. Mol. Cell Biol..

[90] Nguyen AT, Zhang Y (2011). The diverse functions of Dot1 and H3K79 methylation. Genes Dev..

[91] Hatanaka Y (2017). Histone H3 Methylated at Arginine 17 Is Essential for Reprogramming the Paternal Genome in Zygotes. Cell Rep..

[92] Du J, Johnson LM, Jacobsen SE, Patel DJ (2015). DNA methylation pathways and their crosstalk with histone methylation. Nat. Rev. Mol. Cell Biol..

[93] Laugesen A, Højfeldt JW, Helin K (2019). Molecular Mechanisms Directing PRC2 Recruitment and H3K27 Methylation. Mol. Cell.

[94] Hayashi-Takanaka Y (2021). Chromatin loading of MCM hexamers is associated with di-/tri-methylation of histone H4K20 toward S phase entry. Nucleic Acids Res.

[95] Blanc RS, Richard S (2017). Arginine Methylation: The Coming of Age. Mol. Cell.

[96] Swatek KN, Komander D (2016). Ubiquitin modifications. Cell Res.

[97] Mattiroli F, Penengo L (2021). Histone Ubiquitination: An Integrative Signaling Platform in Genome Stability. Trends Genet.

[98] Walser F (2020). Ubiquitin Phosphorylation at Thr12 Modulates the DNA Damage Response. Mol. Cell.

[99] Worden EJ, Hoffmann NA, Hicks CW, Wolberger C (2019). Mechanism of Cross-talk between H2B Ubiquitination and H3 Methylation by Dot1L. Cell.

[100] Nowak SJ, Corces VG (2004). Phosphorylation of histone H3: a balancing act between chromosome condensation and transcriptional activation. Trends Genet.

[101] Jeffery NN (2021). Histone H2A.X phosphorylation and Caspase-Initiated Chromatin Condensation in late-stage erythropoiesis. Epigenet. Chromatin.

[102] Dong Q, Han F (2012). Phosphorylation of histone H2A is associated with centromere function and maintenance in meiosis. Plant J..

[103] Xie Z (2016). Metabolic Regulation of Gene Expression by Histone Lysine β-Hydroxybutyrylation. Mol. Cell.

[104] Zhou T (2022). Function and mechanism of histone β-hydroxybutyrylation in health and disease. Front Immunol..

[105] Chen Y (2007). Lysine propionylation and butyrylation are novel post-translational modifications in histones. Mol. Cell Proteom..

[106] Sabari BR (2015). Intracellular crotonyl-CoA stimulates transcription through p300-catalyzed histone crotonylation. Mol. Cell.

[107] Zhang D (2019). Metabolic regulation of gene expression by histone lactylation. Nature.

[108] Parmar JJ, Padinhateeri R (2020). Nucleosome positioning and chromatin organization. Curr. Opin. Struct. Biol..

[109] Lorch Y, Kornberg RD (2017). Chromatin-remodeling for transcription. Q Rev. Biophys..

[110] Cenik BK, Shilatifard A (2021). COMPASS and SWI/SNF complexes in development and disease. Nat. Rev. Genet.

[111] Li Y (2021). The emerging role of ISWI chromatin remodeling complexes in cancer. J. Exp. Clin. Cancer Res.

[112] Liu C, Kang N, Guo Y, Gong P (2021). Advances in Chromodomain Helicase DNA-Binding (CHD) Proteins Regulating Stem Cell Differentiation and Human Diseases. Front Cell Dev. Biol..

[113] Willhoft O, Wigley DB (2020). INO80 and SWR1 complexes: the non-identical twins of chromatin remodelling. Curr. Opin. Struct. Biol..

[114] Han Y, Reyes AA, Malik S, He Y (2020). Cryo-EM structure of SWI/SNF complex bound to a nucleosome. Nature.

[115] Clapier CR, Iwasa J, Cairns BR, Peterson CL (2017). Mechanisms of action and regulation of ATP-dependent chromatin-remodelling complexes. Nat. Rev. Mol. Cell Biol..

[116] Reyes AA, Marcum RD, He Y (2021). Structure and Function of Chromatin Remodelers. J. Mol. Biol..

[117] Iyer MK (2015). The landscape of long noncoding RNAs in the human transcriptome. Nat. Genet.

[118] St Laurent G, Wahlestedt C, Kapranov P (2015). The Landscape of long noncoding RNA classification. Trends Genet.

[119] Sharp PA (2009). The centrality of RNA. Cell.

[120] Ling H, Fabbri M, Calin GA (2013). MicroRNAs and other non-coding RNAs as targets for anticancer drug development. Nat. Rev. Drug Disco..

[121] Liu C (2019). Long non-coding RNA LINC01207 silencing suppresses AGR2 expression to facilitate autophagy and apoptosis of pancreatic cancer cells by sponging miR-143-5p. Mol. Cell Endocrinol..

[122] Liu L (2019). Circular RNA ciRS-7 promotes the proliferation and metastasis of pancreatic cancer by regulating miR-7-mediated EGFR/STAT3 signaling pathway. Hepatobi. Pancreat. Dis. Int.

[123] Kuo FC (2022). HOTAIR interacts with PRC2 complex regulating the regional preadipocyte transcriptome and human fat distribution. Cell Rep..

[124] Landthaler M, Yalcin A, Tuschl T (2004). The human DiGeorge syndrome critical region gene 8 and Its D. melanogaster homolog are required for miRNA biogenesis. Curr. Biol..

[125] Bartel DP (2009). MicroRNAs: target recognition and regulatory functions. Cell.

[126] Yao Q, Chen Y, Zhou X (2019). The roles of microRNAs in epigenetic regulation. Curr. Opin. Chem. Biol..

[127] Poddar S, Kesharwani D, Datta M (2017). Interplay between the miRNome and the epigenetic machinery: Implications in health and disease. J. Cell Physiol..

[128] Jiang H (2021). miRNA-204-5p acts as tumor suppressor to influence the invasion and migration of astrocytoma by targeting ezrin and is downregulated by DNA methylation. Bioengineered.

[129] Ji C, Guo X (2019). The clinical potential of circulating microRNAs in obesity. Nat. Rev. Endocrinol..

[130] Huang, J. P. et al. Exosomal microRNAs miR-30d-5p and miR-126a-5p Are Associated with Heart Failure with Preserved Ejection Fraction in STZ-Induced Type 1 Diabetic Rats. *Int. J. Mol. Sci*. **23**, 7514 (2022). 10.3390/ijms23147514 PMC9318774 35886860

[131] Quinn JJ, Chang HY (2016). Unique features of long non-coding RNA biogenesis and function. Nat. Rev. Genet.

[132] Geisler S, Coller J (2013). RNA in unexpected places: long non-coding RNA functions in diverse cellular contexts. Nat. Rev. Mol. Cell Biol..

[133] Zhao Z (2020). Mechanisms of lncRNA/microRNA interactions in angiogenesis. Life Sci..

[134] Huang W (2022). LncRNA-mediated DNA methylation: an emerging mechanism in cancer and beyond. J. Exp. Clin. Cancer Res.

[135] Sun TT (2016). LncRNA GClnc1 Promotes Gastric Carcinogenesis and May Act as a Modular Scaffold of WDR5 and KAT2A Complexes to Specify the Histone Modification Pattern. Cancer Disco..

[136] Yang Y (2020). The roles of miRNA, lncRNA and circRNA in the development of osteoporosis. Biol. Res.

[137] Dieter C (2021). The Impact of lncRNAs in Diabetes Mellitus: A Systematic Review and In Silico Analyses. Front Endocrinol. (Lausanne).

[138] Kristensen LS (2019). The biogenesis, biology and characterization of circular RNAs. Nat. Rev. Genet.

[139] Li Z (2015). Exon-intron circular RNAs regulate transcription in the nucleus. Nat. Struct. Mol. Biol..

[140] Schreiner S, Didio A, Hung LH, Bindereif A (2020). Design and application of circular RNAs with protein-sponge function. Nucleic Acids Res.

[141] Dong ZR (2021). CircMEMO1 modulates the promoter methylation and expression of TCF21 to regulate hepatocellular carcinoma progression and sorafenib treatment sensitivity. Mol. Cancer.

[142] Jie M (2020). CircMRPS35 suppresses gastric cancer progression via recruiting KAT7 to govern histone modification. Mol. Cancer.

[143] Zeng Y, Zheng Z, Liu F, Yi G (2021). Circular RNAs in metabolism and metabolic disorders. Obes. Rev..

[144] Ling C (2008). Epigenetic regulation of PPARGC1A in human type 2 diabetic islets and effect on insulin secretion. Diabetologia.

[145] Barrès R (2009). Non-CpG methylation of the PGC-1alpha promoter through DNMT3B controls mitochondrial density. Cell Metab..

[146] Yang BT (2011). Insulin promoter DNA methylation correlates negatively with insulin gene expression and positively with HbA(1c) levels in human pancreatic islets. Diabetologia.

[147] Volkmar M (2012). DNA methylation profiling identifies epigenetic dysregulation in pancreatic islets from type 2 diabetic patients. Embo. j..

[148] Dayeh T (2014). Genome-wide DNA methylation analysis of human pancreatic islets from type 2 diabetic and non-diabetic donors identifies candidate genes that influence insulin secretion. PLoS Genet.

[149] Orozco LD (2018). Epigenome-wide association in adipose tissue from the METSIM cohort. Hum. Mol. Genet.

[150] Krause C (2020). Multi-layered epigenetic regulation of IRS2 expression in the liver of obese individuals with type 2 diabetes. Diabetologia.

[151] García-Calzón, S. et al. Epigenetic markers associated with metformin response and intolerance in drug-naïve patients with type 2 diabetes. *Sci Transl Med*. **12**, eaaz1803 (2020). 10.1126/scitranslmed.aaz1803 32938793

[152] Juvinao-Quintero DL (2021). DNA methylation of blood cells is associated with prevalent type 2 diabetes in a meta-analysis of four European cohorts. Clin. Epigenet..

[153] Yang L (2018). Effect of TET2 on the pathogenesis of diabetic nephropathy through activation of transforming growth factor β1 expression via DNA demethylation. Life Sci..

[154] Yang S (2022). Genome-wide DNA methylation analysis of extreme phenotypes in the identification of novel epigenetic modifications in diabetic retinopathy. Clin. Epigenet..

[155] Zhao J (2021). Transient High Glucose Causes Persistent Vascular Dysfunction and Delayed Wound Healing by the DNMT1-Mediated Ang-1/NF-κB Pathway. J. Invest Dermatol.

[156] Sayols-Baixeras S (2017). DNA methylation and obesity traits: An epigenome-wide association study. The REGICOR study. Epigenetics.

[157] Wahl S (2017). Epigenome-wide association study of body mass index, and the adverse outcomes of adiposity. Nature.

[158] Demerath EW (2015). Epigenome-wide association study (EWAS) of BMI, BMI change and waist circumference in African American adults identifies multiple replicated loci. Hum. Mol. Genet.

[159] Dhana K (2018). An Epigenome-Wide Association Study of Obesity-Related Traits. Am. J. Epidemiol..

[160] Aslibekyan S (2015). Epigenome-wide study identifies novel methylation loci associated with body mass index and waist circumference. Obes. (Silver Spring).

[161] Mendelson MM (2017). Association of Body Mass Index with DNA Methylation and Gene Expression in Blood Cells and Relations to Cardiometabolic Disease: A Mendelian Randomization Approach. PLoS Med.

[162] Trayhurn P (2013). Hypoxia and adipose tissue function and dysfunction in obesity. Physiol. Rev..

[163] Kayser B, Verges S (2021). Hypoxia, energy balance, and obesity: An update. Obes. Rev..

[164] Dick KJ (2014). DNA methylation and body-mass index: a genome-wide analysis. Lancet.

[165] Wang S (2015). HIF3A DNA Methylation Is Associated with Childhood Obesity and ALT. PLoS One.

[166] Bollepalli S (2018). Subcutaneous adipose tissue gene expression and DNA methylation respond to both short- and long-term weight loss. Int J. Obes. (Lond.).

[167] Keller M (2020). DNA methylation signature in blood mirrors successful weight-loss during lifestyle interventions: the CENTRAL trial. Genome Med.

[168] Perfilyev A (2017). Impact of polyunsaturated and saturated fat overfeeding on the DNA-methylation pattern in human adipose tissue: a randomized controlled trial. Am. J. Clin. Nutr..

[169] Kadayifci, F. Z., Zheng, S. & Pan, Y. X. Molecular Mechanisms Underlying the Link between Diet and DNA Methylation. *Int J Mol Sci*. **19**, (2018). 10.3390/ijms19124055 PMC6320837 30558203

[170] Chen HC, Chen YZ, Wang CH, Lin FJ (2020). The nonalcoholic fatty liver disease-like phenotype and lowered serum VLDL are associated with decreased expression and DNA hypermethylation of hepatic ApoB in male offspring of ApoE deficient mothers fed a with Western diet. J. Nutr. Biochem.

[171] Baumeier C (2017). Elevated hepatic DPP4 activity promotes insulin resistance and non-alcoholic fatty liver disease. Mol. Metab..

[172] Baumeier C (2017). Hepatic DPP4 DNA Methylation Associates With Fatty Liver. Diabetes.

[173] Hosseini H (2020). Resveratrol alleviates non-alcoholic fatty liver disease through epigenetic modification of the Nrf2 signaling pathway. Int J. Biochem Cell Biol..

[174] Walle P (2016). Fatty acid metabolism is altered in non-alcoholic steatohepatitis independent of obesity. Metabolism.

[175] Walle P (2019). Liver DNA methylation of FADS2 associates with FADS2 genotype. Clin. Epigenet..

[176] Lai Z (2020). Association of Hepatic Global DNA Methylation and Serum One-Carbon Metabolites with Histological Severity in Patients with NAFLD. Obes. (Silver Spring).

[177] Cheishvili D (2018). Identification of an Epigenetic Signature of Osteoporosis in Blood DNA of Postmenopausal Women. J. Bone Min. Res.

[178] Raje MM, Ashma R (2019). Epigenetic regulation of BMP2 gene in osteoporosis: a DNA methylation study. Mol. Biol. Rep..

[179] Fernandez-Rebollo E (2018). Primary Osteoporosis Is Not Reflected by Disease-Specific DNA Methylation or Accelerated Epigenetic Age in Blood. J. Bone Min. Res.

[180] Ono T, Hayashi M, Sasaki F, Nakashima T (2020). RANKL biology: bone metabolism, the immune system, and beyond. Inflamm. Regen..

[181] Wang P (2018). Influence of DNA methylation on the expression of OPG/RANKL in primary osteoporosis. Int J. Med Sci..

[182] Wang JS, Mazur CM, Wein MN (2021). Sclerostin and Osteocalcin: Candidate Bone-Produced Hormones. Front Endocrinol. (Lausanne).

[183] Reppe S (2015). Methylation of bone SOST, its mRNA, and serum sclerostin levels correlate strongly with fracture risk in postmenopausal women. J. Bone Min. Res.

[184] Shan Y (2019). Methylation of bone SOST impairs SP7, RUNX2, and ERα transactivation in patients with postmenopausal osteoporosis. Biochem Cell Biol..

[185] Cao Y (2019). Expression of Sclerostin in Osteoporotic Fracture Patients Is Associated with DNA Methylation in the CpG Island of the SOST Gene. Int J. Genom..

[186] Hu Q (2020). Histone Deacetylase 3 Aggravates Type 1 Diabetes Mellitus by Inhibiting Lymphocyte Apoptosis Through the microRNA-296-5p/Bcl-xl Axis. Front Genet.

[187] Zhang L (2021). CBP/p300 HAT maintains the gene network critical for β cell identity and functional maturity. Cell Death Dis..

[188] Xu Z (2021). METTL14-regulated PI3K/Akt signaling pathway via PTEN affects HDAC5-mediated epithelial-mesenchymal transition of renal tubular cells in diabetic kidney disease. Cell Death Dis..

[189] Du W (2019). STAT3 phosphorylation mediates high glucose-impaired cell autophagy in an HDAC1-dependent and -independent manner in Schwann cells of diabetic peripheral neuropathy. Faseb j..

[190] Che S, Wu S, Yu P (2022). Downregulated HDAC3 or up-regulated microRNA-296-5p alleviates diabetic retinopathy in a mouse model. Regen. Ther..

[191] denDekker AD (2020). TNF-α regulates diabetic macrophage function through the histone acetyltransferase MOF. JCI Insight.

[192] Pessoa Rodrigues C (2021). Histone H4 lysine 16 acetylation controls central carbon metabolism and diet-induced obesity in mice. Nat. Commun..

[193] Cricrí D (2021). Histone Deacetylase 3 Regulates Adipocyte Phenotype at Early Stages of Differentiation. Int J Mol Sci.

[194] Qian H (2017). HDAC6-mediated acetylation of lipid droplet-binding protein CIDEC regulates fat-induced lipid storage. J. Clin. Invest.

[195] Lieber AD (2019). Loss of HDAC6 alters gut microbiota and worsens obesity. Faseb j..

[196] Sun L (2018). Programming and Regulation of Metabolic Homeostasis by HDAC11. EBioMedicine.

[197] Bagchi RA (2018). HDAC11 suppresses the thermogenic program of adipose tissue via BRD2. JCI Insight.

[198] Wang T (2021). Acetylation of lactate dehydrogenase B drives NAFLD progression by impairing lactate clearance. J. Hepatol..

[199] Hu MJ, Long M, Dai RJ (2022). Acetylation of H3K27 activated lncRNA NEAT1 and promoted hepatic lipid accumulation in non-alcoholic fatty liver disease via regulating miR-212-5p/GRIA3. Mol. Cell Biochem.

[200] Zhou B (2020). The Nuclear Orphan Receptor NR2F6 Promotes Hepatic Steatosis through Upregulation of Fatty Acid Transporter CD36. Adv. Sci. (Weinh.)..

[201] Zhong S (2017). Cluster of Differentiation 36 Deficiency Aggravates Macrophage Infiltration and Hepatic Inflammation by Upregulating Monocyte Chemotactic Protein-1 Expression of Hepatocytes Through Histone Deacetylase 2-Dependent Pathway. Antioxid. Redox Signal.

[202] Zhang L (2021). S100A11 Promotes Liver Steatosis via FOXO1-Mediated Autophagy and Lipogenesis. Cell Mol. Gastroenterol. Hepatol..

[203] Fu R (2020). Endothelial ZEB1 promotes angiogenesis-dependent bone formation and reverses osteoporosis. Nat. Commun..

[204] Lian WS (2019). MicroRNA-29a represses osteoclast formation and protects against osteoporosis by regulating PCAF-mediated RANKL and CXCL12. Cell Death Dis..

[205] Zhang P (2016). Histone H3K9 Acetyltransferase PCAF Is Essential for Osteogenic Differentiation Through Bone Morphogenetic Protein Signaling and May Be Involved in Osteoporosis. Stem Cells.

[206] Ma C (2021). HDAC6 inactivates Runx2 promoter to block osteogenesis of bone marrow stromal cells in age-related bone loss of mice. Stem Cell Res Ther..

[207] Hu M (2022). NAP1L2 drives mesenchymal stem cell senescence and suppresses osteogenic differentiation. Aging Cell.

[208] Wang J (2016). Mechanical stimulation orchestrates the osteogenic differentiation of human bone marrow stromal cells by regulating HDAC1. Cell Death Dis..

[209] Zhang P (2016). Histone Acetyltransferase GCN5 Regulates Osteogenic Differentiation of Mesenchymal Stem Cells by Inhibiting NF-κB. J. Bone Min. Res.

[210] Kimball AS (2019). The Histone Methyltransferase Setdb2 Modulates Macrophage Phenotype and Uric Acid Production in Diabetic Wound Repair. Immunity.

[211] Gao Y, Ge W (2018). The histone methyltransferase DOT1L inhibits osteoclastogenesis and protects against osteoporosis. Cell Death Dis..

[212] Bricambert J (2018). The histone demethylase Phf2 acts as a molecular checkpoint to prevent NAFLD progression during obesity. Nat. Commun..

[213] Alghamdi TA (2018). Histone H3 Serine 10 Phosphorylation Facilitates Endothelial Activation in Diabetic Kidney Disease. Diabetes.

[214] Najafova Z (2021). RNF40 exerts stage-dependent functions in differentiating osteoblasts and is essential for bone cell crosstalk. Cell Death Differ..

[215] Wang C (2020). PARP1 Hinders Histone H2B Occupancy at the NFATc1 Promoter to Restrain Osteoclast Differentiation. J. Bone Min. Res.

[216] Liao W (2022). Persistent high glucose induced EPB41L4A-AS1 inhibits glucose uptake via GCN5 mediating crotonylation and acetylation of histones and non-histones. Clin. Transl. Med.

[217] Filippakopoulos P, Knapp S (2014). Targeting bromodomains: epigenetic readers of lysine acetylation. Nat. Rev. Drug Disco..

[218] Kaeser MD (2008). BRD7, a novel PBAF-specific SWI/SNF subunit, is required for target gene activation and repression in embryonic stem cells. J. Biol. Chem..

[219] Kadoch C (2013). Proteomic and bioinformatic analysis of mammalian SWI/SNF complexes identifies extensive roles in human malignancy. Nat. Genet.

[220] Wei Z (2018). Vitamin D Switches BAF Complexes to Protect β Cells. Cell.

[221] Spaeth JM (2019). The Pdx1-Bound Swi/Snf Chromatin Remodeling Complex Regulates Pancreatic Progenitor Cell Proliferation and Mature Islet β-Cell Function. Diabetes.

[222] Kong, Q. et al. BAF60a Deficiency in Macrophage Promotes Diet-Induced Obesity and Metabolic Inflammation. *Diabetes*, (2022). 10.2337/db22-0114 35822944

[223] Meng ZX (2013). Baf60c drives glycolytic metabolism in the muscle and improves systemic glucose homeostasis through Deptor-mediated Akt activation. Nat. Med.

[224] Sen S (2017). Transcription factor 19 interacts with histone 3 lysine 4 trimethylation and controls gluconeogenesis via the nucleosome-remodeling-deacetylase complex. J. Biol. Chem..

[225] Li S (2008). Genome-wide coactivation analysis of PGC-1alpha identifies BAF60a as a regulator of hepatic lipid metabolism. Cell Metab..

[226] Meng ZX (2015). A Diet-Sensitive BAF60a-Mediated Pathway Links Hepatic Bile Acid Metabolism to Cholesterol Absorption and Atherosclerosis. Cell Rep..

[227] Wang Y (2013). Phosphorylation and recruitment of BAF60c in chromatin remodeling for lipogenesis in response to insulin. Mol. Cell.

[228] Zhou C, Zou J, Zou S, Li X (2016). INO80 is Required for Osteogenic Differentiation of Human Mesenchymal Stem Cells. Sci. Rep..

[229] Nguyen KH (2015). SWI/SNF-Mediated Lineage Determination in Mesenchymal Stem Cells Confers Resistance to Osteoporosis. Stem Cells.

[230] Xie Y (2022). Melatonin enhances osteoblastogenesis of senescent bone marrow stromal cells through NSD2-mediated chromatin remodelling. Clin. Transl. Med.

[231] Tian J (2020). NF-κB inhibits the occurrence of type 1 diabetes through microRNA-150-dependent PUMA degradation. Life Sci..

[232] Ofori JK (2022). Human Islet MicroRNA-200c Is Elevated in Type 2 Diabetes and Targets the Transcription Factor ETV5 to Reduce Insulin Secretion. Diabetes.

[233] McIntyre HD (2019). Gestational diabetes mellitus. Nat. Rev. Dis. Prim..

[234] Ye, Z. et al. Plasma exosomal microRNAs Associated with Metabolism as Early Predictor of Gestational Diabetes Mellitus. *Diabetes*, (2022). 10.2337/db21-0909 PMC9630082 35926094

[235] Guan CY (2022). miR-199a Is Upregulated in GDM Targeting the MeCP2-Trpc3 Pathway. Front Endocrinol. (Lausanne).

[236] Zhang Y (2022). MicroRNA-146a-5p-modified human umbilical cord mesenchymal stem cells enhance protection against diabetic nephropathy in rats through facilitating M2 macrophage polarization. Stem Cell Res Ther..

[237] Hu J (2022). Resveratrol Enhances Wound Healing in Type 1 Diabetes Mellitus by Promoting the Expression of Extracellular Vesicle-Carried MicroRNA-129 Derived from Mesenchymal Stem Cells. J. Proteome Res.

[238] Zhang R (2022). A Potential Target for Diabetic Vascular Damage: High Glucose-Induced Monocyte Extracellular Vesicles Impair Endothelial Cells by Delivering miR-142-5p. Front Bioeng. Biotechnol..

[239] Zhang D (2021). Upregulation of Mir342 in Diet-Induced Obesity Mouse and the Hypothalamic Appetite Control. Front Endocrinol. (Lausanne).

[240] Gao Y (2019). MicroRNA miR-7 and miR-17-92 in the Arcuate Nucleus of Mouse Hypothalamus Regulate Sex-Specific Diet-Induced Obesity. Mol. Neurobiol..

[241] Tryggestad JB (2019). Macrophage-Derived microRNA-155 Increases in Obesity and Influences Adipocyte Metabolism by Targeting Peroxisome Proliferator-Activated Receptor Gamma. Obes. (Silver Spring).

[242] Ying W (2021). MiR-690, an exosomal-derived miRNA from M2-polarized macrophages, improves insulin sensitivity in obese mice. Cell Metab..

[243] Pan Y (2019). Adipocyte-secreted exosomal microRNA-34a inhibits M2 macrophage polarization to promote obesity-induced adipose inflammation. J. Clin. Invest.

[244] Huang XY (2022). Exosomal miR-122 promotes adipogenesis and aggravates obesity through the VDR/SREBF1 axis. Obes. (Silver Spring).

[245] Chang J (2008). Liver-specific microRNA miR-122 enhances the replication of hepatitis C virus in nonhepatic cells. J. Virol..

[246] Long JK, Dai W, Zheng YW, Zhao S (2019). P. miR-122 promotes hepatic lipogenesis via inhibiting the LKB1/AMPK pathway by targeting Sirt1 in non-alcoholic fatty liver disease. Mol. Med.

[247] Lee YH (2021). Hepatic MIR20B promotes nonalcoholic fatty liver disease by suppressing PPARA. Elife.

[248] Hou X (2021). Myeloid-Cell-Specific IL-6 Signaling Promotes MicroRNA-223-Enriched Exosome Production to Attenuate NAFLD-Associated Fibrosis. Hepatology.

[249] Zhang T (2019). MicroRNA-378 promotes hepatic inflammation and fibrosis via modulation of the NF-κB-TNFα pathway. J. Hepatol..

[250] Lee DH (2021). Mir214-3p and Hnf4a/Hnf4α reciprocally regulate Ulk1 expression and autophagy in nonalcoholic hepatic steatosis. Autophagy.

[251] Xu H (2021). An Endoplasmic Reticulum Stress-MicroRNA-26a Feedback Circuit in NAFLD. Hepatology.

[252] Dai Z, Wei G (2022). Inhibition of miRNA-100 facilitates bone regeneration defects of mesenchymal stem cells in osteoporotic mice through the protein kinase B pathway. Bioengineered.

[253] Li S (2022). MiR-152-5p suppresses osteogenic differentiation of mandible mesenchymal stem cells by regulating ATG14-mediated autophagy. Stem Cell Res Ther..

[254] John AA (2022). AAV-mediated delivery of osteoblast/osteoclast-regulating miRNAs for osteoporosis therapy. Mol. Ther. Nucleic Acids.

[255] Hu L (2022). MiR-1224-5p modulates osteogenesis by coordinating osteoblast/osteoclast differentiation via the Rap1 signaling target ADCY2. Exp. Mol. Med.

[256] Zhang, D., Du, J., Yu, M. & Suo, L. Urine-derived stem cells-extracellular vesicles ameliorate diabetic osteoporosis through HDAC4/HIF-1α/VEGFA axis by delivering microRNA-26a-5p. *Cell Biol Toxicol*, (2022). 10.1007/s10565-022-09713-5 35554780

[257] Behera, J., Ison, J., Voor, M. J. & Tyagi, N. Exercise-Linked Skeletal Irisin Ameliorates Diabetes-Associated Osteoporosis by Inhibiting the Oxidative Damage-Dependent miR-150-FNDC5/Pyroptosis Axis. *Diabetes*, (2022). 10.2337/db21-0573 PMC9750954 35802043

[258] You M (2022). Bone mesenchymal stem cells (BMSCs)-derived exosomal microRNA-21-5p regulates Kruppel-like factor 3 (KLF3) to promote osteoblast proliferation in vitro. Bioengineered.

[259] Liu LL (2022). ATF1/miR-214-5p/ITGA7 axis promotes osteoclastogenesis to alter OVX-induced bone absorption. Mol. Med.

[260] Huang YN (2021). Long, Noncoding RNA SRA Induces Apoptosis of β-Cells by Promoting the IRAK1/LDHA/Lactate Pathway. Int J Mol Sci.

[261] Chen J (2018). Long noncoding RNA MALAT1 regulates generation of reactive oxygen species and the insulin responses in male mice. Biochem Pharm..

[262] Cao Q (2022). Long-noncoding RNA HOXA transcript at the distal tip ameliorates the insulin resistance and hepatic gluconeogenesis in mice with gestational diabetes mellitus via the microRNA-423-5p/wingless-type MMTV integration site family member 7A axis. Bioengineered.

[263] Song P (2022). LncRNA MALAT1 Aggravates Renal Tubular Injury via Activating LIN28A and the Nox4/AMPK/mTOR Signaling Axis in Diabetic Nephropathy. Front Endocrinol. (Lausanne).

[264] Han T, Li W, Zhang H, Nie D (2022). Involvement of long non-coding RNA ZNF503 antisense RNA 1 in diabetic retinopathy and its possible underlying mechanism. Bioengineered.

[265] Yu P (2022). lncRNA-H19 in Fibroblasts Promotes Wound Healing in Diabetes. Diabetes.

[266] Zhang T (2020). The lncRNA RP11-142A22.4 promotes adipogenesis by sponging miR-587 to modulate Wnt5β expression. Cell Death Dis..

[267] Xu H (2021). Lnc13728 facilitates human mesenchymal stem cell adipogenic differentiation via positive regulation of ZBED3 and downregulation of the WNT/β-catenin pathway. Stem Cell Res Ther..

[268] Li K (2019). H19/miR-30a/C8orf4 axis modulates the adipogenic differentiation process in human adipose tissue-derived mesenchymal stem cells. J. Cell Physiol..

[269] Xiao F (2021). Long Non-coding RNA 332443 Inhibits Preadipocyte Differentiation by Targeting Runx1 and p38-MAPK and ERK1/2-MAPK Signaling Pathways. Front Cell Dev. Biol..

[270] Zhang L (2022). LncRNA MIR99AHG enhances adipocyte differentiation by targeting miR-29b-3p to upregulate PPARγ. Mol. Cell Endocrinol..

[271] Chen J (2017). The role and possible mechanism of lncRNA U90926 in modulating 3T3-L1 preadipocyte differentiation. Int J. Obes. (Lond.).

[272] Wu C (2022). Long noncoding RNA XIST regulates brown preadipocytes differentiation and combats high-fat diet induced obesity by targeting C/EBPα. Mol. Med.

[273] Wang Y (2020). The Effect of FOXC2-AS1 on White Adipocyte Browning and the Possible Regulatory Mechanism. Front Endocrinol. (Lausanne).

[274] Ma M (2020). The lncRNA Gm15622 stimulates SREBP-1c expression and hepatic lipid accumulation by sponging the miR-742-3p in mice. J. Lipid Res.

[275] Jiang Y (2021). Loss of Hilnc prevents diet-induced hepatic steatosis through binding of IGF2BP2. Nat. Metab..

[276] Leti F (2017). Altered expression of MALAT1 lncRNA in nonalcoholic steatohepatitis fibrosis regulates CXCL5 in hepatic stellate cells. Transl. Res.

[277] Yu F (2017). NEAT1 accelerates the progression of liver fibrosis via regulation of microRNA-122 and Kruppel-like factor 6. J. Mol. Med (Berl.).

[278] Shen X, Guo H, Xu J, Wang J (2019). Inhibition of lncRNA HULC improves hepatic fibrosis and hepatocyte apoptosis by inhibiting the MAPK signaling pathway in rats with nonalcoholic fatty liver disease. J. Cell Physiol..

[279] Ye L (2021). LncRNA-Gm9795 promotes inflammation in non-alcoholic steatohepatitis via NF-[Formula: see text]B/JNK pathway by endoplasmic reticulum stress. J. Transl. Med.

[280] Lin Y (2021). Oscillating lncRNA Platr4 regulates NLRP3 inflammasome to ameliorate nonalcoholic steatohepatitis in mice. Theranostics.

[281] Wang F (2022). LncRNA MIAT can regulate the proliferation, apoptosis, and osteogenic differentiation of bone marrow-derived mesenchymal stem cells by targeting miR-150-5p. Bioengineered.

[282] Li, B. et al. LncRNA RAD51-AS1 Regulates Human Bone Marrow Mesenchymal Stem Cells via Interaction with YBX1 to Ameliorate Osteoporosis. *Stem Cell Rev Rep*, (2022). 10.1007/s12015-022-10408-x 35727431

[283] Yang Y (2021). Exosome-Derived LncRNA TCONS_00072128 Mediated Osteogenic Differentiation and Inflammation by Caspase 8 Regulation. Front Genet.

[284] Han Y (2022). Epigallocatechin gallate attenuates tumor necrosis factor (TNF)-α-induced inhibition of osteoblastic differentiation by up-regulating lncRNA TUG1 in osteoporosis. Bioengineered.

[285] Yin C (2022). Long noncoding RNA Lnc-DIF inhibits bone formation by sequestering miR-489-3p. iScience.

[286] Liu C (2022). The mechanosensitive lncRNA Neat1 promotes osteoblast function through paraspeckle-dependent Smurf1 mRNA retention. Bone Res.

[287] Ren L (2022). Inflammatory osteoclasts-derived exosomes promote bone formation by selectively transferring lncRNA LIOCE into osteoblasts to interact with and stabilize Osterix. Faseb j..

[288] Yang Z (2022). Bioactive glass nanoparticles inhibit osteoclast differentiation and osteoporotic bone loss by activating lncRNA NRON expression in the extracellular vesicles derived from bone marrow mesenchymal stem cells. Biomaterials.

[289] Liu C (2019). LncRNA AK077216 promotes RANKL-induced osteoclastogenesis and bone resorption via NFATc1 by inhibition of NIP45. J. Cell Physiol..

[290] Yan YX (2022). CircRNA hsa_circ_0071336 is associated with type 2 diabetes through targeting the miR-93-5p/GLUT4 axis. Faseb j..

[291] Zhang C (2020). Circular RNA circPPM1F modulates M1 macrophage activation and pancreatic islet inflammation in type 1 diabetes mellitus. Theranostics.

[292] Du R (2022). circMAP3K4 regulates insulin resistance in trophoblast cells during gestational diabetes mellitus by modulating the miR-6795-5p/PTPN1 axis. J. Transl. Med.

[293] Liu Q (2022). Circ_NNT suppresses the apoptosis and inflammation in glucose-induced human retinal pigment epithelium by regulating miR-320b/TIMP3 axis in diabetic retinopathy. Clin. Immunol..

[294] Jiang J (2022). Circular RNA circHIPK3 is downregulated in diabetic cardiomyopathy and overexpression of circHIPK3 suppresses PTEN to protect cardiomyocytes from high glucose-induced cell apoptosis. Bioengineered.

[295] Meng F (2022). CircARHGAP12 Triggers Mesenchymal Stromal Cell Autophagy to Facilitate its Effect on Repairing Diabetic Wounds by Sponging miR-301b-3p/ATG16L1 and miR-301b-3p/ULK2. J. Invest Dermatol.

[296] Liu Y (2020). Circular RNA SAMD4A controls adipogenesis in obesity through the miR-138-5p/EZH2 axis. Theranostics.

[297] Kang Z (2020). circFLT1 and lncCCPG1 Sponges miR-93 to Regulate the Proliferation and Differentiation of Adipocytes by Promoting lncSLC30A9 Expression. Mol. Ther. Nucleic Acids.

[298] Wu J (2022). CircRNA Profiling Reveals CircPPARγ Modulates Adipogenic Differentiation via Sponging miR-92a-3p. J. Agric Food Chem..

[299] Jiang R (2020). circRNA Profiling Reveals an Abundant circFUT10 that Promotes Adipocyte Proliferation and Inhibits Adipocyte Differentiation via Sponging let-7. Mol. Ther. Nucleic Acids.

[300] Liu K (2022). CircRNA-mediated regulation of brown adipose tissue adipogenesis. Front Nutr..

[301] Arcinas C (2019). Adipose circular RNAs exhibit dynamic regulation in obesity and functional role in adipogenesis. Nat. Metab..

[302] Zhang Z (2019). circARF3 Alleviates Mitophagy-Mediated Inflammation by Targeting miR-103/TRAF3 in Mouse Adipose Tissue. Mol. Ther. Nucleic Acids.

[303] Li J (2021). A nanodrug system overexpressed circRNA_0001805 alleviates nonalcoholic fatty liver disease via miR-106a-5p/miR-320a and ABCA1/CPT1 axis. J. Nanobiotechnol..

[304] Chen X (2021). Circ_0057558 promotes nonalcoholic fatty liver disease by regulating ROCK1/AMPK signaling through targeting miR-206. Cell Death Dis..

[305] Yang W (2020). Hsa_circ_0048179 attenuates free fatty acid-induced steatosis via hsa_circ_0048179/miR-188-3p/GPX4 signaling. Aging (Albany NY).

[306] Zhao Q (2020). Targeting Mitochondria-Located circRNA SCAR Alleviates NASH via Reducing mROS Output. Cell.

[307] Jin X (2020). Antagonizing circRNA_002581-miR-122-CPEB1 axis alleviates NASH through restoring PTEN-AMPK-mTOR pathway regulated autophagy. Cell Death Dis..

[308] Zhang M, Jia L, Zheng Y (2019). circRNA Expression Profiles in Human Bone Marrow Stem Cells Undergoing Osteoblast Differentiation. Stem Cell Rev. Rep..

[309] Ouyang Z (2019). CircRNA hsa_circ_0074834 promotes the osteogenesis-angiogenesis coupling process in bone mesenchymal stem cells (BMSCs) by acting as a ceRNA for miR-942-5p. Cell Death Dis..

[310] Yu L, Liu Y (2019). circRNA_0016624 could sponge miR-98 to regulate BMP2 expression in postmenopausal osteoporosis. Biochem Biophys. Res Commun..

[311] Qiao L, Li CG, Liu D (2020). CircRNA_0048211 protects postmenopausal osteoporosis through targeting miRNA-93-5p to regulate BMP2. Eur. Rev. Med Pharm. Sci..

[312] Chen G (2022). Circular RNA circStag1 promotes bone regeneration by interacting with HuR. Bone Res.

[313] Mi B (2019). CircRNA AFF4 promotes osteoblast cells proliferation and inhibits apoptosis via the Mir-7223-5p/PIK3R1 axis. Aging (Albany NY).

[314] Liang J (2020). Circular RNA HIPK3 downregulation mediates hydrogen peroxide-induced cytotoxicity in human osteoblasts. Aging (Albany NY).

[315] Chen X (2019). CircRNA_28313/miR-195a/CSF1 axis modulates osteoclast differentiation to affect OVX-induced bone absorption in mice. RNA Biol..

[316] Wang Q (2022). Suppression of osteoclast multinucleation via a posttranscriptional regulation-based spatiotemporally selective delivery system. Sci. Adv..

[317] Dalbeth N, Gosling AL, Gaffo A, Abhishek A (2021). Gout. Lancet.

[318] Zhong X (2016). Association of DNA methyltransferase polymorphisms with susceptibility to primary gouty arthritis. Biomed. Rep..

[319] Wang Z (2020). Differential DNA Methylation of Networked Signaling, Transcriptional, Innate and Adaptive Immunity, and Osteoclastogenesis Genes and Pathways in Gout. Arthritis Rheumatol..

[320] Tseng CC (2020). Systemic Investigation of Promoter-wide Methylome and Genome Variations in Gout. Int J Mol Sci.

[321] Li B (2017). CCL2 promoter hypomethylation is associated with gout risk in Chinese Han male population. Immunol. Lett..

[322] Yang Y (2017). Elevated UMOD methylation level in peripheral blood is associated with gout risk. Sci. Rep..

[323] Zhu Z (2017). DNA hypomethylation of a transcription factor binding site within the promoter of a gout risk gene NRBP1 upregulates its expression by inhibition of TFAP2A binding. Clin. Epigenet..

[324] Cleophas MCP (2019). Romidepsin suppresses monosodium urate crystal-induced cytokine production through upregulation of suppressor of cytokine signaling 1 expression. Arthritis Res Ther..

[325] Crișan TO (2016). Soluble uric acid primes TLR-induced proinflammatory cytokine production by human primary cells via inhibition of IL-1Ra. Ann. Rheum. Dis..

[326] Li G (2021). MiR-221-5p is involved in the regulation of inflammatory responses in acute gouty arthritis by targeting IL-1β. Int J. Rheum. Dis..

[327] Zhou W (2017). MicroRNA-488 and -920 regulate the production of proinflammatory cytokines in acute gouty arthritis. Arthritis Res Ther..

[328] Liu YF, Xing GL, Chen Z, Tu SH (2021). Long non-coding RNA HOTAIR knockdown alleviates gouty arthritis through miR-20b upregulation and NLRP3 downregulation. Cell Cycle.

[329] Zhang X (2020). lncRNA‑MM2P downregulates the production of pro‑inflammatory cytokines in acute gouty arthritis. Mol. Med Rep..

[330] Lian C (2021). Circular RNA circHIPK3 Activates Macrophage NLRP3 Inflammasome and TLR4 Pathway in Gouty Arthritis via Sponging miR-561 and miR-192. Inflammation.

[331] Meng Q (2021). Total glucosides of paeony protects THP-1 macrophages against monosodium urate-induced inflammation via MALAT1/miR-876-5p/NLRP3 signaling cascade in gouty arthritis. Biomed. Pharmacother..

[332] De Leo S, Lee SY, Braverman LE (2016). Hyperthyroidism. Lancet.

[333] Limbach M (2016). Epigenetic profiling in CD4+ and CD8+ T cells from Graves’ disease patients reveals changes in genes associated with T cell receptor signaling. J. Autoimmun..

[334] Guo Q (2018). Alterations of Global DNA Methylation and DNA Methyltransferase Expression in T and B Lymphocytes from Patients with Newly Diagnosed Autoimmune Thyroid Diseases After Treatment: A Follow-Up Study. Thyroid.

[335] Hashimoto H (2019). Association of IFNG gene methylation in peripheral blood cells with the development and prognosis of autoimmune thyroid diseases. Cytokine.

[336] Song RH (2021). METTL3 Is Involved in the Development of Graves’ Disease by Inducing SOCS mRNA m6A Modification. Front Endocrinol. (Lausanne).

[337] Virakul S (2020). Integrative Analysis of Proteomics and DNA Methylation in Orbital Fibroblasts From Graves’ Ophthalmopathy. Front Endocrinol. (Lausanne).

[338] Rotondo Dottore G (2021). Genetic Profiling of Orbital Fibroblasts from Patients with Graves’ Orbitopathy. J. Clin. Endocrinol. Metab..

[339] Ekronarongchai S (2021). Histone Deacetylase 4 Controls Extracellular Matrix Production in Orbital Fibroblasts from Graves’ Ophthalmopathy Patients. Thyroid.

[340] Chen X (2018). Serum and thyroid tissue level of let-7b and their correlation with TRAb in Graves’ disease. J. Transl. Med.

[341] Wang N (2021). LncRNA LPAL2/miR-1287-5p/EGFR Axis Modulates TED-Derived Orbital Fibroblast Activation Through Cell Adhesion Factors. J. Clin. Endocrinol. Metab..

[342] Sun Y (2020). Microarray profiling and functional analysis of differentially expressed plasma exosomal circular RNAs in Graves’ disease. Biol. Res.

[343] Chaker L, Bianco AC, Jonklaas J, Peeters RP (2017). Hypothyroidism. Lancet.

[344] Luo J, Wang X, Yuan L, Guo L (2021). Genome‑wide profiling of DNA methylation and gene expression unravel the epigenetic landscape in diabetes-related hypothyroidism. Clin. Epigenet..

[345] Kim DW, Park JW, Willingham MC, Cheng SY (2014). A histone deacetylase inhibitor improves hypothyroidism caused by a TRα1 mutant. Hum. Mol. Genet.

[346] Susetyo A (2022). Histone Deacetylase 3 Inhibitor Alleviates Cerebellar Defects in Perinatal Hypothyroid Mice by Stimulating Histone Acetylation and Transcription at Thyroid Hormone-Responsive Gene Loci. Int J Mol Sci.

[347] Tokić S (2018). miR-29a-3p/T-bet Regulatory Circuit Is Altered in T Cells of Patients With Hashimoto’s Thyroiditis. Front Endocrinol. (Lausanne).

[348] Wang C (2020). Rno-miR-224-5p contributes to 2,2’,4,4’-tetrabromodiphenyl ether-induced low triiodothyronine in rats by targeting deiodinases. Chemosphere.

[349] Xiu L (2018). miRNA-125b-5p Suppresses Hypothyroidism Development by Targeting Signal Transducer and Activator of Transcription 3. Med Sci. Monit..

[350] Wang L (2021). Long non-coding RNA NEAT1 regulates endothelial functions in subclinical hypothyroidism through miR-126/TRAF7 pathway. Hum. Cell.

[351] Horvath S (2013). DNA methylation age of human tissues and cell types. Genome Biol..

[352] Ling C (2007). Genetic and epigenetic factors are associated with expression of respiratory chain component NDUFB6 in human skeletal muscle. J. Clin. Invest.

[353] Rönn T (2008). Age influences DNA methylation and gene expression of COX7A1 in human skeletal muscle. Diabetologia.

[354] Bacos K (2016). Blood-based biomarkers of age-associated epigenetic changes in human islets associate with insulin secretion and diabetes. Nat. Commun..

[355] Wang R (2020). Epigenetic Regulation in Mesenchymal Stem Cell Aging and Differentiation and Osteoporosis. Stem Cells Int.

[356] Kowluru RA (2020). Retinopathy in a Diet-Induced Type 2 Diabetic Rat Model and Role of Epigenetic Modifications. Diabetes.

[357] Georgel PT, Georgel P (2021). Where Epigenetics Meets Food Intake: Their Interaction in the Development/Severity of Gout and Therapeutic Perspectives. Front Immunol..

[358] Nitert MD (2012). Impact of an exercise intervention on DNA methylation in skeletal muscle from first-degree relatives of patients with type 2 diabetes. Diabetes.

[359] Poursafa P (2022). DNA methylation: a potential mediator between air pollution and metabolic syndrome. Clin. Epigenet..

[360] Dayeh TA (2013). Identification of CpG-SNPs associated with type 2 diabetes and differential DNA methylation in human pancreatic islets. Diabetologia.

[361] Olsson AH (2014). Genome-wide associations between genetic and epigenetic variation influence mRNA expression and insulin secretion in human pancreatic islets. PLoS Genet.

[362] Shah UJ (2019). Differential methylation of the type 2 diabetes susceptibility locus KCNQ1 is associated with insulin sensitivity and is predicted by CpG site specific genetic variation. Diabetes Res Clin. Pr..

[363] Grundberg E (2013). Global analysis of DNA methylation variation in adipose tissue from twins reveals links to disease-associated variants in distal regulatory elements. Am. J. Hum. Genet.

[364] Nilsson E (2014). Altered DNA methylation and differential expression of genes influencing metabolism and inflammation in adipose tissue from subjects with type 2 diabetes. Diabetes.

[365] Dayeh T (2016). DNA methylation of loci within ABCG1 and PHOSPHO1 in blood DNA is associated with future type 2 diabetes risk. Epigenetics.

[366] Chambers JC (2015). Epigenome-wide association of DNA methylation markers in peripheral blood from Indian Asians and Europeans with incident type 2 diabetes: a nested case-control study. Lancet Diabetes Endocrinol..

[367] Johnson ND (2021). Differential DNA methylation and changing cell-type proportions as fibrotic stage progresses in NAFLD. Clin. Epigenet..

[368] Pirola CJ (2013). Epigenetic modification of liver mitochondrial DNA is associated with histological severity of nonalcoholic fatty liver disease. Gut.

[369] Guay SP (2014). ADRB3 gene promoter DNA methylation in blood and visceral adipose tissue is associated with metabolic disturbances in men. Epigenomics.

[370] Pezzolesi MG (2015). Circulating TGF-β1-Regulated miRNAs and the Risk of Rapid Progression to ESRD in Type 1 Diabetes. Diabetes.

[371] Mostahfezian M, Azhir Z, Dehghanian F, Hojati Z (2019). Expression Pattern of microRNAs, miR-21, miR-155 and miR-338 in Patients with Type 1 Diabetes. Arch. Med Res.

[372] La Sala L (2019). Circulating microRNA-21 is an early predictor of ROS-mediated damage in subjects with high risk of developing diabetes and in drug-naïve T2D. Cardiovasc Diabetol..

[373] Miao L (2019). Circulating miR-3659 may be a potential biomarker of dyslipidemia in patients with obesity. J. Transl. Med.

[374] Erhartova D (2019). Serum miR-33a is associated with steatosis and inflammation in patients with non-alcoholic fatty liver disease after liver transplantation. PLoS One.

[375] Jiang H (2021). Circulating microRNA-135a-3p in serum extracellular vesicles as a potential biological marker of non-alcoholic fatty liver disease. Mol Med Rep.

[376] Al-Rawaf HA, Alghadir AH, Gabr SA (2021). Circulating MicroRNA Expression, Vitamin D, and Hypercortisolism as Predictors of Osteoporosis in Elderly Postmenopausal Women. Dis. Markers.

[377] Han S (2020). Circular RNA hsa_circ_0076690 acts as a prognostic biomarker in osteoporosis and regulates osteogenic differentiation of hBMSCs via sponging miR-152. Aging (Albany NY).

[378] Chang TT (2022). Antioxidation and Nrf2-mediated heme oxygenase-1 activation contribute to renal protective effects of hydralazine in diabetic nephropathy. Biomed. Pharmacother..

[379] Larkin, B. P. et al. Low-dose hydralazine reduces albuminuria and glomerulosclerosis in a mouse model of obesity-related chronic kidney disease. *Diabetes Obes Metab*, (2022). 10.1111/dom.14778 PMC9544807 35635331

[380] Lee BH, Yegnasubramanian S, Lin X, Nelson WG (2005). Procainamide is a specific inhibitor of DNA methyltransferase 1. J. Biol. Chem..

[381] Bernard H (2020). Coxsackievirus B Type 4 Infection in β Cells Downregulates the Chaperone Prefoldin URI to Induce a MODY4-like Diabetes via Pdx1 Silencing. Cell Rep. Med.

[382] Gao J (2019). Decitabine assists umbilical cord-derived mesenchymal stem cells in improving glucose homeostasis by modulating macrophage polarization in type 2 diabetic mice. Stem Cell Res Ther..

[383] Wang X (2016). Epigenetic regulation of macrophage polarization and inflammation by DNA methylation in obesity. JCI Insight.

[384] Li YY (2018). Fatty liver mediated by peroxisome proliferator-activated receptor-α DNA methylation can be reversed by a methylation inhibitor and curcumin. J. Dig. Dis..

[385] Li Y (2020). Advanced glycation end products inhibit the osteogenic differentiation potential of adipose-derived stem cells by modulating Wnt/β-catenin signalling pathway via DNA methylation. Cell Prolif..

[386] Khan S, Jena G (2016). Valproic Acid Improves Glucose Homeostasis by Increasing Beta-Cell Proliferation, Function, and Reducing its Apoptosis through HDAC Inhibition in Juvenile Diabetic Rat. J. Biochem Mol. Toxicol..

[387] Lin JR (2021). Valproic Acid Suppresses Autoimmune Recurrence and Allograft Rejection in Islet Transplantation through Induction of the Differentiation of Regulatory T Cells and Can Be Used in Cell Therapy for Type 1 Diabetes. Pharmaceuticals (Basel).

[388] Schroeder TM, Westendorf JJ (2005). Histone deacetylase inhibitors promote osteoblast maturation. J. Bone Min. Res.

[389] McGee-Lawrence ME (2018). Loss of Hdac3 in osteoprogenitors increases bone expression of osteoprotegerin, improving systemic insulin sensitivity. J. Cell Physiol..

[390] Advani A (2011). Long-term administration of the histone deacetylase inhibitor vorinostat attenuates renal injury in experimental diabetes through an endothelial nitric oxide synthase-dependent mechanism. Am. J. Pathol..

[391] Gilbert RE (2011). Histone deacetylase inhibition attenuates diabetes-associated kidney growth: potential role for epigenetic modification of the epidermal growth factor receptor. Kidney Int.

[392] Ma J (2021). SAHA induces white fat browning and rectifies metabolic dysfunctions via activation of ZFPs. J. Endocrinol..

[393] Dudakovic A (2013). Histone deacetylase inhibition promotes osteoblast maturation by altering the histone H4 epigenome and reduces Akt phosphorylation. J. Biol. Chem..

[394] Ozcan U (2006). Chemical chaperones reduce ER stress and restore glucose homeostasis in a mouse model of type 2 diabetes. Science.

[395] Xiao C, Giacca A, Lewis GF (2011). Sodium phenylbutyrate, a drug with known capacity to reduce endoplasmic reticulum stress, partially alleviates lipid-induced insulin resistance and beta-cell dysfunction in humans. Diabetes.

[396] Lewis EC (2011). The oral histone deacetylase inhibitor ITF2357 reduces cytokines and protects islet β cells in vivo and in vitro. Mol. Med.

[397] Christensen DP (2014). Lysine deacetylase inhibition prevents diabetes by chromatin-independent immunoregulation and β-cell protection. Proc. Natl Acad. Sci. USA.

[398] Besançon A (2018). Oral histone deacetylase inhibitor synergises with T cell targeted immunotherapy to preserve beta cell metabolic function and induce stable remission of new-onset autoimmune diabetes in NOD mice. Diabetologia.

[399] Huang HM (2022). Histone deacetylase inhibitor givinostat attenuates nonalcoholic steatohepatitis and liver fibrosis. Acta Pharm. Sin..

[400] Chu XY (2021). Identification of Dacinostat as a potential anti-obesity compound through transcriptional activation of adipose thermogenesis in mice. Biochim Biophys. Acta Mol. Basis Dis..

[401] Lv X (2021). HDAC inhibitor Trichostatin A suppresses adipogenesis in 3T3-L1 preadipocytes. Aging (Albany NY).

[402] Olaniyi KS, Amusa OA (2020). Sodium acetate-mediated inhibition of histone deacetylase alleviates hepatic lipid dysregulation and its accompanied injury in streptozotocin-nicotinamide-induced diabetic rats. Biomed. Pharmacother..

[403] Guo CJ (2019). Puerarin alleviates streptozotocin (STZ)-induced osteoporosis in rats through suppressing inflammation and apoptosis via HDAC1/HDAC3 signaling. Biomed. Pharmacother..

[404] Nelson KM (2017). The Essential Medicinal Chemistry of Curcumin. J. Med Chem..

[405] Ren BC (2020). Curcumin alleviates oxidative stress and inhibits apoptosis in diabetic cardiomyopathy via Sirt1-Foxo1 and PI3K-Akt signalling pathways. J. Cell Mol. Med.

[406] Tikoo K, Meena RL, Kabra DG, Gaikwad AB (2008). Change in post-translational modifications of histone H3, heat-shock protein-27 and MAP kinase p38 expression by curcumin in streptozotocin-induced type I diabetic nephropathy. Br. J. Pharm..

[407] Wang Y (2015). Novel curcumin analog C66 prevents diabetic nephropathy via JNK pathway with the involvement of p300/CBP-mediated histone acetylation. Biochim Biophys. Acta.

[408] Peng J (2022). The P300 acetyltransferase inhibitor C646 promotes membrane translocation of insulin receptor protein substrate and interaction with the insulin receptor. J. Biol. Chem..

[409] Bagul PK, Deepthi N, Sultana R, Banerjee SK (2015). Resveratrol ameliorates cardiac oxidative stress in diabetes through deacetylation of NFkB-p65 and histone 3. J. Nutr. Biochem.

[410] Fang WJ (2018). Resveratrol alleviates diabetic cardiomyopathy in rats by improving mitochondrial function through PGC-1α deacetylation. Acta Pharm. Sin..

[411] Lagouge M (2006). Resveratrol improves mitochondrial function and protects against metabolic disease by activating SIRT1 and PGC-1alpha. Cell.

[412] Wang X (2017). Resveratrol ameliorates hyperglycemia-induced renal tubular oxidative stress damage via modulating the SIRT1/FOXO3a pathway. Diabetes Res Clin. Pr..

[413] Jiang Y (2020). Resveratrol promotes osteogenesis via activating SIRT1/FoxO1 pathway in osteoporosis mice. Life Sci..

[414] Liu S, Fang Y, Yu J, Chang X (2021). Hawthorn polyphenols reduce high glucose-induced inflammation and apoptosis in ARPE-19 cells by regulating miR-34a/SIRT1 to reduce acetylation. J. Food Biochem.

[415] Milne JC (2007). Small molecule activators of SIRT1 as therapeutics for the treatment of type 2 diabetes. Nature.

[416] Morgan ES (2019). Antisense Inhibition of Glucagon Receptor by IONIS-GCGR(Rx) Improves Type 2 Diabetes Without Increase in Hepatic Glycogen Content in Patients With Type 2 Diabetes on Stable Metformin Therapy. Diabetes Care.

[417] Gaudet D (2020). Vupanorsen, an N-acetyl galactosamine-conjugated antisense drug to ANGPTL3 mRNA, lowers triglycerides and atherogenic lipoproteins in patients with diabetes, hepatic steatosis, and hypertriglyceridaemia. Eur. Heart J..

[418] Yuan X (2015). Berberine ameliorates nonalcoholic fatty liver disease by a global modulation of hepatic mRNA and lncRNA expression profiles. J. Transl. Med.

[419] Gjaltema RAF, Rots MG (2020). Advances of epigenetic editing. Curr. Opin. Chem. Biol..

[420] Thakore PI, Black JB, Hilton IB, Gersbach CA (2016). Editing the epigenome: technologies for programmable transcription and epigenetic modulation. Nat. Methods.

[421] Ou K (2019). Targeted demethylation at the CDKN1C/p57 locus induces human β cell replication. J. Clin. Invest.

[422] Liao HK (2017). In Vivo Target Gene Activation via CRISPR/Cas9-Mediated Trans-epigenetic Modulation. Cell.

[423] Syding LA, Nickl P, Kasparek P, Sedlacek R (2020). CRISPR/Cas9 Epigenome Editing Potential for Rare Imprinting Diseases: A Review. Cells.

[424] Claussnitzer M (2015). FTO Obesity Variant Circuitry and Adipocyte Browning in Humans. N. Engl. J. Med.

[425] Altucci L, Rots MG (2016). Epigenetic drugs: from chemistry via biology to medicine and back. Clin. Epigenetics.

